# Orthogonal Design of Competing Deprotonation Process in Cation‐Mediated Ni(OH)_2_ for Achieving Industrial Level Biomass Electrooxidation

**DOI:** 10.1002/advs.75458

**Published:** 2026-04-27

**Authors:** Junge Yang, Zhengjie Chen, Lili Zhang, Tao Zhang, Shida Bao, Jing Peng, Hui Pan, Hui‐Ming Cheng

**Affiliations:** ^1^ Institute of Technology for Carbon Neutrality. Shenzhen Key Laboratory of Energy Materials for Carbon Neutrality Shenzhen Institute of Advanced Technology Chinese Academy of Sciences Shenzhen China; ^2^ Institute of Applied Physics and Materials Engineering University of Macau Macao SAR China; ^3^ Faculty of Materials Science and Energy Engineering Shenzhen University of Advanced Technology Shenzhen China; ^4^ Department of Physics and Chemistry Faculty of Science and Technology University of Macau Macao SAR China; ^5^ Shenyang National Laboratory for Materials Science Institute of Metal Research Chinese Academy of Sciences Shenyang China

**Keywords:** biomass upgrading, competitive reaction, HMF electrooxidation, industrial current density, orthogonal activity

## Abstract

The electrooxidation of 5‐hydroxymethylfurfural (HMF) to 2,5‐furandicarboxylic acid (FDCA) has attracted great attention in biomass value‐added conversion. However, the central challenge for achieving industrial‐level scalable biomass upgrading is that the catalysts’ inherent preference for activating O─H bond in the competitive oxygen evolution reaction (OER) over the strong C─H bond in organic molecules. Herein, we designed a Cu‐substituted Ni(OH)_2_ catalyst with orthogonal activity by precise valence engineering, selectively activating the aldehyde C─H in HMF oxidation while remaining inert toward the O─H cleavage in OER. Mechanistic studies and in situ characterizations confirm that the incorporation of Cu creates a unique local environment that fine‐tunes the deprotonation kinetics in two competing reactions, thereby improving selectivity and activity. This specific orthogonal design delivers outstanding performance, achieving an industrial‐scale current density of 1 A cm^−2^ at 1.55 V vs. RHE with a high Faraday efficiency for FDCA of 99.5%, which is comparable to the best catalysts reported to date. More importantly, the catalyst is continuously stable over 15 cycles. This work provides a new strategy for designing advanced electrocatalysts to achieve selective biomass conversion under industrial‐scale conditions.

## Graphical abstract

A Cu‐mediated Ni(OH)_2_ catalyst with orthogonal activity by precise valence engineering simultaneously accelerates rate‐determining aldehyde C─H activation in HMF oxidation and impedes O─H deprotonation in competing OER. This multi‐functional synergistic catalysis enables FDCA production at industrial‐level current densities without sacrificing FE_FDCA_ and selectivity_FDCA_.

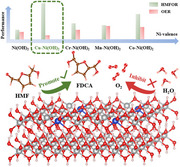

## 1 Introduction

The electrochemical conversion of 5‐hydroxymethylfurfural (HMF) into value‐added 2,5‐furandicarboxylic acid (FDCA) is a critical path to sustainable chemical manufacturing [1, 2, 3, 4]. Recently, the HMF electrooxidation reaction (HMFOR) has shown distinct advantages over traditional thermocatalytic methods, including milder operation conditions [5, 6], faster reaction kinetics [7, 8], and the potential for direct integration with renewable electricity sources [9, 10, 11]. Besides, it is an alternative to the sluggish anodic oxygen evolution reaction (OER) of water electrolysis due to its ideal theoretical onset potential (0.113 V vs. RHE) [12, 13]. However, the complete 6‐electron oxidation of HMF to FDCA requires sequential conversion of alcohol (─CH_2_OH) and aldehyde (─CHO) groups to carboxylic acids(─COOH) [14, 15, 16]. While most electrocatalysts (e.g., Ni‐based (oxy)hydroxides) can efficiently oxidize alcohols by a hydride transfer mechanism [17], the aldehyde oxidation step is a severe kinetic barrier [18, 19]. It proceeds through the aldehyde hydrate intermediate (R‐CH(OH)O^−^ in alkaline media), whose deprotonation requires the cleavage of a strong C─H bond adjacent to the carbonyl group (R‐CH(OH)O^−^ → R‐C(O)OH + H^+^ + 2e^−^) [20]. The intrinsically high dissociation energy of this C─H bond imposes a large activation barrier [21]. On conventional NiOOH surfaces, this barrier makes the aldehyde‐to‐acid transformation the rate‐determining step (RDS) [22, 23], ultimately limiting FDCA partial current densities even under high applied potentials.

A further challenge arises from the competing oxygen evolution reaction (OER), which consumes both electrons and catalytic sites. The OER mechanism on NiOOH involves sequential deprotonation steps of adsorbed oxygen intermediates (^*^OH, ^*^O, ^*^OOH) [2, 24, 25]. The initial deprotonation of ^*^OH (^*^OH → ^*^O + H^+^ + e^−^) requires breaking a strong metal‐adsorbate O─H bond [26, 27], which is thermodynamically well aligned with the potential window for HMF oxidation, because the ^*^OH binding energy on Ni^3+^ sites favors the cleavage of the O─H bond [28, 29, 30]. As a result, when the applied potential is increased to increase current density, the kinetically easy O─H bond cleavage in OER intermediates readily outcompetes the much slower aldehyde C─H cleavage in HMFOR [31]. This competition dramatically reduces the Faraday efficiency (FE) for FDCA, especially under the conditions required for industrial current densities.

Therefore, accelerating the sluggish aldehyde‐to‐acid conversion while suppressing the competing OER is essential for the efficient HMFOR at high current densities [32]. This necessitates moving beyond conventional monometallic catalysts to more complex designs exploiting multifunctional effects. Strategies such as incorporating bi‐ or tri‐metallic alloys [33], coupling metals with oxides [34], and introducing metal‐hydroxide interfaces [35] can generate new active sites or change the electronic environment of existing ones. However, most reported approaches improve either HMF oxidation or OER suppression, but rarely both. This underscores the need for catalysts that integrate multiple functions to give a balanced performance.

Herein, we report a Ni‐valence engineering by Cu‐substituted Ni(OH)_2_ catalyst, which achieves efficient HMF electrooxidation with industrial‐level current densities. Ni(OH)_2_ nanosheets incorporating different metal cations were grown on nickel foam (NF) (denoted as M‐Ni(OH)_2_@NF, where M = Cr, Mn, Co, Cu), which showed different modulation of Ni oxidation states and electronic structures. Of these, Cu‐Ni(OH)_2_@NF has the highest intrinsic activity, reaching current densities of 1000 mA cm^−2^ at 1.55 V vs. RHE and 1200 mA cm^−2^ at 1.60 V vs. RHE in an alkaline electrolyte containing 50 mm HMF. Density functional theory (DFT) calculations, supported by in situ characterizations, show that this outstanding performance arises from easier aldehyde C─H activation during HMFOR, accompanied by weakened O─H bond cleavage in the competing OER. Product analysis confirms an impressive catalytic performance with a high FE_FDCA_ of 99.5% at 1.45 V, and outstanding stability in long‐term electrooxidation tests. This orthogonal design enables the catalyst to sustain exceptionally high current densities without compromising selectivity or durability, marking an important step toward the practical electrochemical production of FDCA from biomass.

## 2 Results and Discussion

### 2.1 Morphology and Structure Characterizations

**FIGURE 1:**
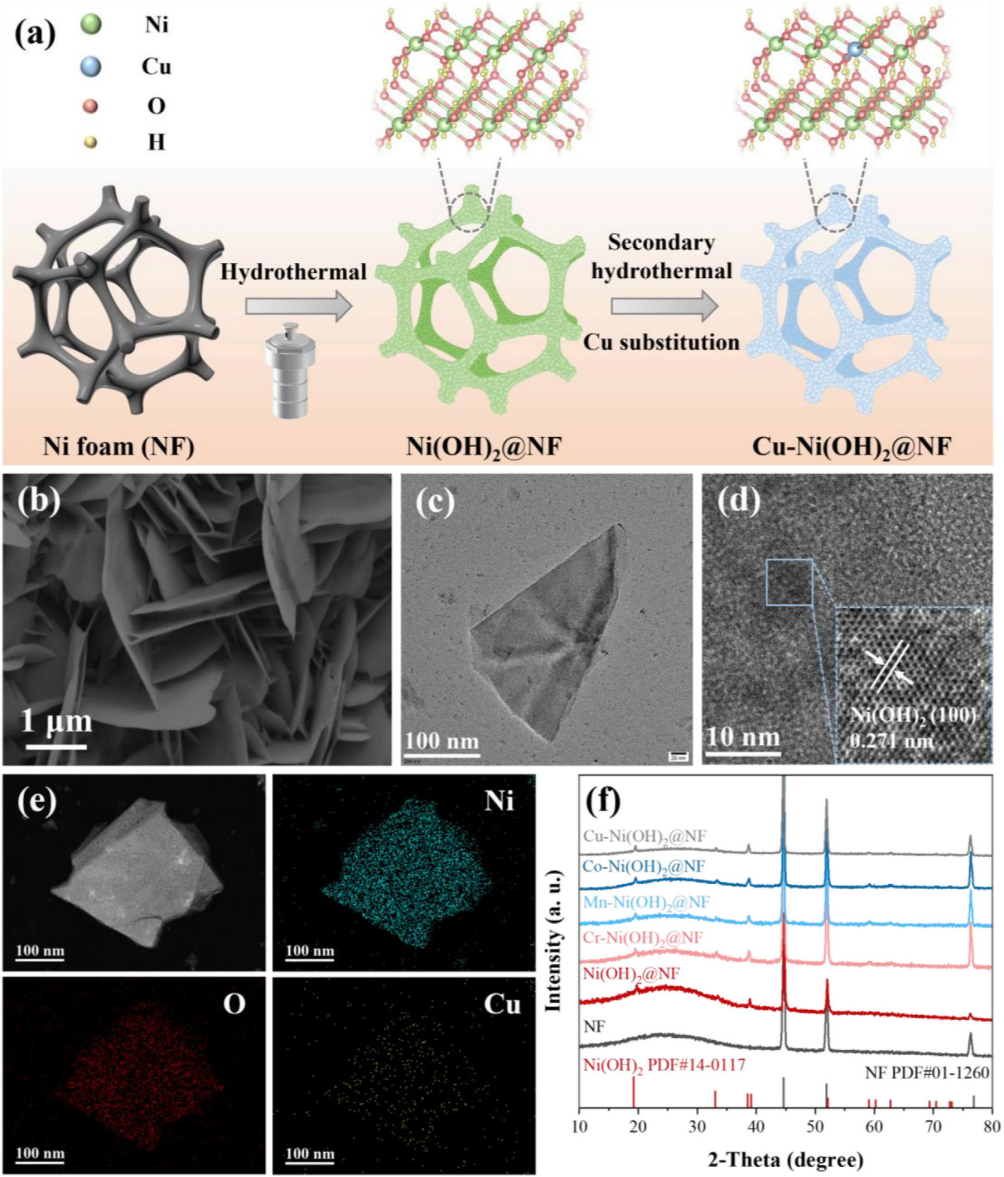
Synthesis and characterization of the catalysts. (a) Schematic of the synthesis of Cu‐Ni(OH)_2_@NF. (b–f) Morphology characterization of Cu‐Ni(OH)_2_@NF. (b) SEM image; (c) low‐resolution TEM; (d) HRTEM image; (e) HAADF image and EDS elemental mappings of Ni, O, and Cu; and (f) XRD patterns of NF, Ni(OH)_2_@NF, and M‐Ni(OH)_2_@NF (M = Cr, Mn, Co, Cu).

The Cu‐Ni(OH)_2_@NF catalyst with a three‐dimensional interconnected nanosheet structure was prepared using a two‐step hydrothermal process. First, Ni(OH)_2_@NF was synthesized as a precursor, followed by Cu substitution (Figure 1a). For comparison, analogous catalysts substituted with Cr, Mn, and Co were also prepared under identical conditions (details provided in Experimental Section). The morphology of Cu‐Ni(OH)_2_@NF was investigated using a field‐emission scanning electron microscope (FE‐SEM). The two‐step hydrothermal treatment produced perpendicularly aligned Cu‐Ni(OH)_2_ nanosheets on the surface of NF, significantly increasing the electroactive surface area (Figure 1b). Transmission electron microscopy (TEM) confirmed that the nanosheets remained intact and well dispersed after ultrasonic exfoliation (Figure 1c). High‐resolution TEM (HRTEM) images showed a lattice spacing of 0.271 nm, corresponding to the (100) plane of Ni(OH)_2_, with a slight lattice expansion attributed to Cu incorporation (Figure 1d). Similar lattice features were observed for catalysts substituted with the other elements (Figure S1), with slight differences depending on the element and its content. High‐angle annular dark‐field scanning TEM (HAADF‐STEM) combined with energy‐dispersive spectroscopy (EDS) confirmed the presence of a low Cu content and the uniform distribution of Ni, O, and Cu across the nanosheets (Figure 1e). Inductively coupled plasma optical emission spectroscopy (ICP‐OES) quantified the Cu content as 4.37 wt.% (Table S1). X‐ray diffraction (XRD) patterns showed that all the catalysts matched well with hexagonal Ni(OH)_2_ (PDF 14–0117) and nickel foam (PDF 01–1206), without any discernible peaks corresponding to the substituted metal oxides or hydroxides (Figure 1f).

**FIGURE 2:**
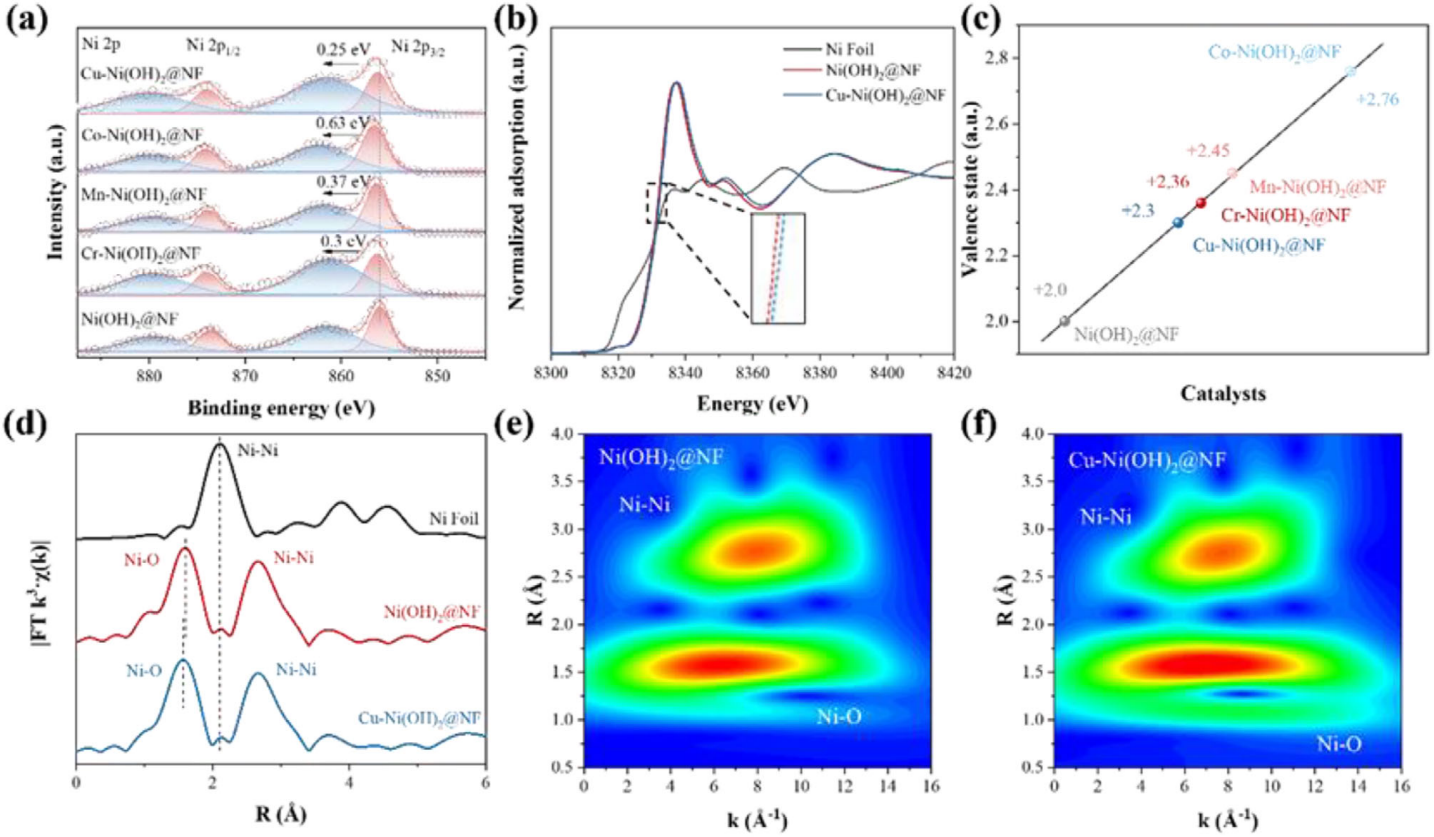
Structural characterizations of M‐Ni(OH)_2_@NF. (a) Ni 2p XPS spectra of Ni(OH)_2_@NF and M‐Ni(OH)_2_@NF; (b) normalized XANES spectra of the Ni K‐edge; (c) average valence state of Ni in different catalysts; (d) FT‐EXAFS spectra of the Ni K‐edge; (e,f) contour plots of the WT of Ni K‐edge EXAFS for (e) Ni(OH)_2_@NF and (f) Cu‐Ni(OH)_2_@NF.

X‐ray photoelectron spectroscopy (XPS) was employed to investigate the effect of the substitution of different metals on the electronic structure and chemical valence of the Ni sites (Figures S2 and S3). In the case of Ni 2p, the two main peaks at 855.92 and 873.78 eV are assigned to Ni^2+^ on the surface Ni(OH)_2_ [36, 37], accompanied by two satellite peaks at 861.56 and 879.58 eV (Figure 2a). Compared with pristine Ni(OH)_2_@NF, the substituted M‐Ni(OH)_2_@NF materials have a systematic positive shift in Ni^2+^ binding energy, in the order: +0.63 eV (Co) > +0.37 eV (Mn) > +0.30 eV (Cr) > +0.25 eV (Cu). This implies that the substitution alters the local electronic structure, thereby inducing multiple valence states in Ni the sites [38]. Among these substitutional elements, the Ni^2+^ peak in Co‐Ni(OH)_2_@NF has the largest shift, indicating that it is in a more oxidizable state, while that in Cu‐Ni(OH)_2_@NF is in a lower oxidation state.

X‐ray absorption spectroscopy (XAS) at the Ni K‐edge was used to elucidate the valence state and local coordination structure of Ni in the Cu‐Ni(OH)_2_@NF. The X‐ray absorption near‐edge structure (XANES) reveals that the absorption edge position for Cu‐Ni(OH)_2_@NF is slightly higher than that of Ni(OH)_2_@NF (Figure 2b), indicating that the introduction of Cu improves the oxidation state of Ni [39], in agreement with the XPS results. A calculation based on K‐edge adsorption energy (Figure S4) shows that the Ni sites in Cu‐Ni(OH)_2_@NF have an average valence state of approximately + 2.30 by considering linear combination fitting of Ni^0^ (Ni foil) and Ni^2+^ (Ni(OH)_2_@NF) [40]. Consistent with the Ni 2p XPS analysis (Figure 2c), the Ni sites in Cu‐Ni(OH)_2_@NF have an intermediate oxidation state compared with the pure hydroxide. Fourier‐transform extended X‐ray absorption fine structure (FT‐EXAFS) analysis provides further insight into the local coordination (Figure 2d). Both Ni(OH)_2_@NF and Cu‐Ni(OH)_2_@NF exhibit characteristic peaks at ∼1.60 Å (Ni─O first shell), ∼2.10 Å (single‐scattering of Ni─Ni), and ∼2.65 Å (second‐shell of Ni─Ni). While the overall coordination motifs remain unchanged, the average Ni─O bond distance reduces by 0.05 Å and the Ni─Ni bond distance increases by 0.02 Å (Figure S5), suggesting definitive lattice distortion induced by the incorporation of Cu [41]. Wavelet transform (WT) analysis of the Ni K‐edge EXAFS distinguishes the scattering contributions (Figure 2e,f). Two dominant intensity regions are observed, corresponding to Ni─O and Ni─Ni bonds. Compared with Ni(OH)_2_@NF, the Ni─O feature in Cu‐Ni(OH)_2_@NF shifts from 6.19 to 6.95 Å^−1^, reflecting a local structural change caused by the Cu substitution.

### 2.2 Electrocatalytic HMFOR Performance

**FIGURE 3:**
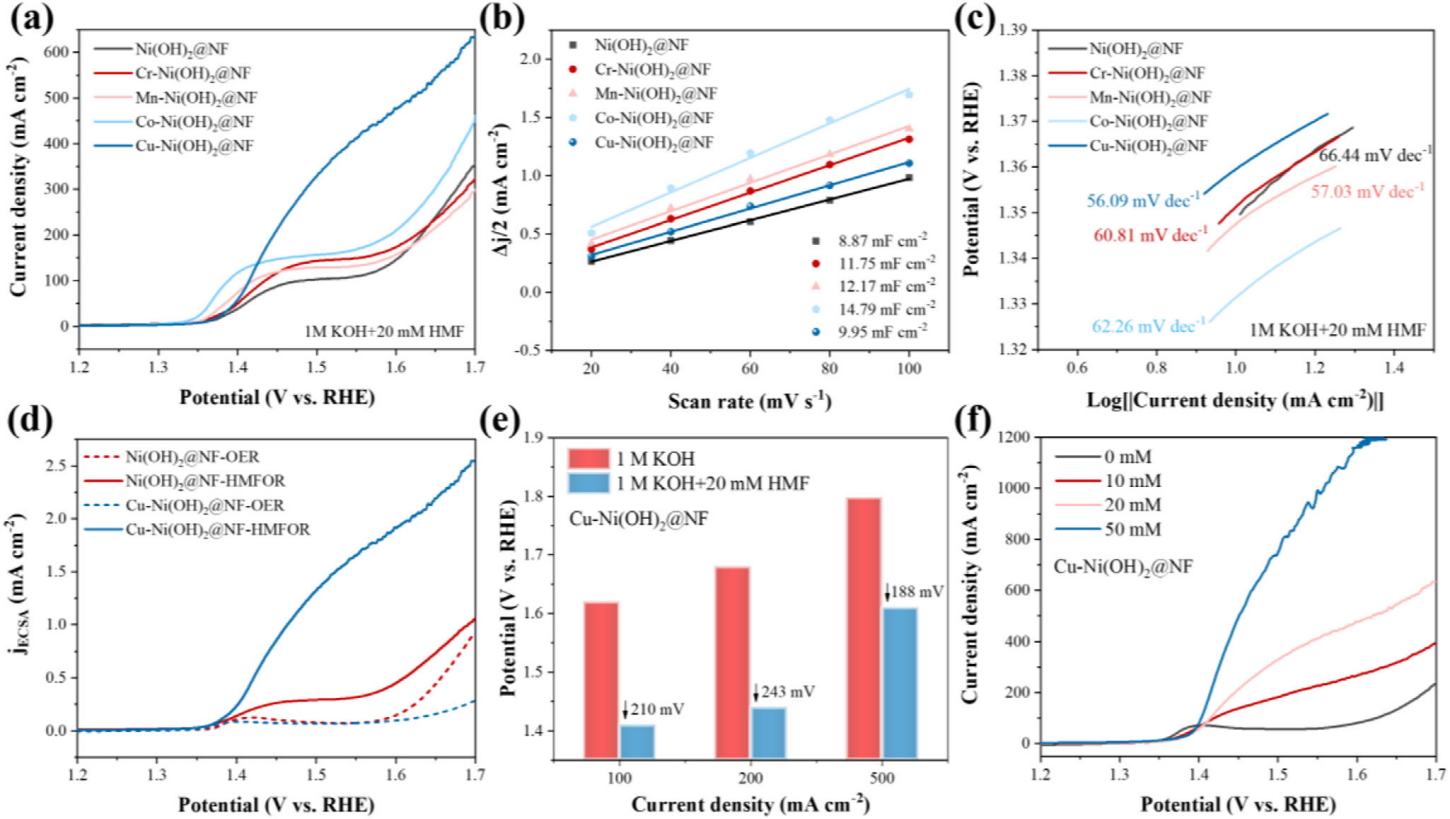
Electrocatalytic performance tests. (a) LSV polarization curves of Ni(OH)_2_@NF and M‐Ni(OH)_2_@NF (M = Cr, Mn, Co, Cu) in the KOH solution with 20 mm HMF; (b) C_dl_ of Ni(OH)_2_@NF and M‐Ni(OH)_2_@NF; (c) corresponding Tafel slopes; (d) ECSA normalized LSV curves of Ni(OH)_2_@NF and Cu‐Ni(OH)_2_@NF with and without 20 mm HMF; (e) potentials for achieving the corresponding current densities of Cu‐Ni(OH)_2_@NF with and without 20 mm HMF; and (f) LSV curves of Cu‐Ni(OH)_2_@NF with different concentrations of HMF.

The electrochemical performance of the catalysts was evaluated in 1 m KOH with 20 mm HMF, using a typical three‐electrode divided H‐type electrochemical cell system. The linear sweep voltammetry (LSV) polarization curves (Figure 3a) indicate that the substituted elements effectively promote HMF electrooxidation performance of Ni(OH)_2_@NF, with Cu‐Ni(OH)_2_@NF achieving the highest current density of 218 mA cm^−2^ at 1.45 V vs. RHE. The M‐Ni(OH)_2_@NF electrocatalysts have different onset potentials for HMFOR, all of which are lower than that of Ni(OH)_2_@NF. Notably, these potentials exhibit a strong correlation with the binding energy shift of the Ni 2p peaks in XPS analysis (Figure 2a), indicating that the substitution‐induced modulation of the valence state at Ni sites has a substantial influence on the HMFOR activation potential. The electrochemical active surface area (ECSA) was evaluated from the double‐layer capacitance (C_dl_), which was derived from cyclic voltammetry (CV) at different scan rates (Figure S6) [42]. The C_dl_ (Figure 3b) values of M‐Ni(OH)_2_@NF are larger than that of Ni(OH)_2_@NF, indicating that the substitution treatment increases the number of exposed active sites. The intrinsic activity is an important indicator of catalytic performance. The LSV curves normalized by ECSA (Figure S7) show that Cu‐Ni(OH)_2_@NF still exhibits the highest current density of all the electrocatalysts, despite possessing less active phase, confirming the superior intrinsic activity of each active sites in Cu‐Ni(OH)_2_@NF. We infer that excessively high valence states accelerate the competitive OER path, while lower valence states result in less significant improvement of HMFOR. Therefore, precise control of the Ni valence state is crucial for determining the overall electrocatalytic efficiency. The corresponding Tafel slopes were calculated from the polarization curves, with Cu‐Ni(OH)_2_@NF having the lowest Tafel slope of 56.09 mV dec^−1^, suggesting faster reaction kinetics than the other catalysts (Figure 3c).

We also analyzed the normalized LSV curves of Cu‐Ni(OH)_2_@NF and Ni(OH)_2_@NF in 1 m KOH with and without 20 mm HMF (Figure 3d; Figure S8). The onset potential of OER is 1.55 V vs. RHE for Cu‐Ni(OH)_2_@NF, and after adding HMF, it decreases to 1.37 V vs. RHE. Ni(OH)_2_@NF shows a worse HMFOR performance due to its relatively high OER activity, especially in the high potential range. The incorporation of Cu effectively suppresses the competitive OER, enhancing HMF electrooxidation. For Cu‐Ni(OH)_2_@NF, the potentials required to reach 100, 200, and 500 mA cm^−2^ are lowered by over 188 mV relative to OER (Figure 3e). At a higher HMF concentration of 50 mm, Cu‐Ni(OH)_2_@NF achieves 1000 mA cm^−2^ at 1.55 V vs. RHE and 1200 mA cm^−2^ at 1.60 V vs. RHE (Figure 3f). Overall, the catalyst's performance surpasses that of most previously reported transition metal‐based electrocatalysts (Table S2), with both a high activity and effective suppression of OER [4, 32].

### 2.3 Exploration of the Catalytic Mechanism

**FIGURE 4:**
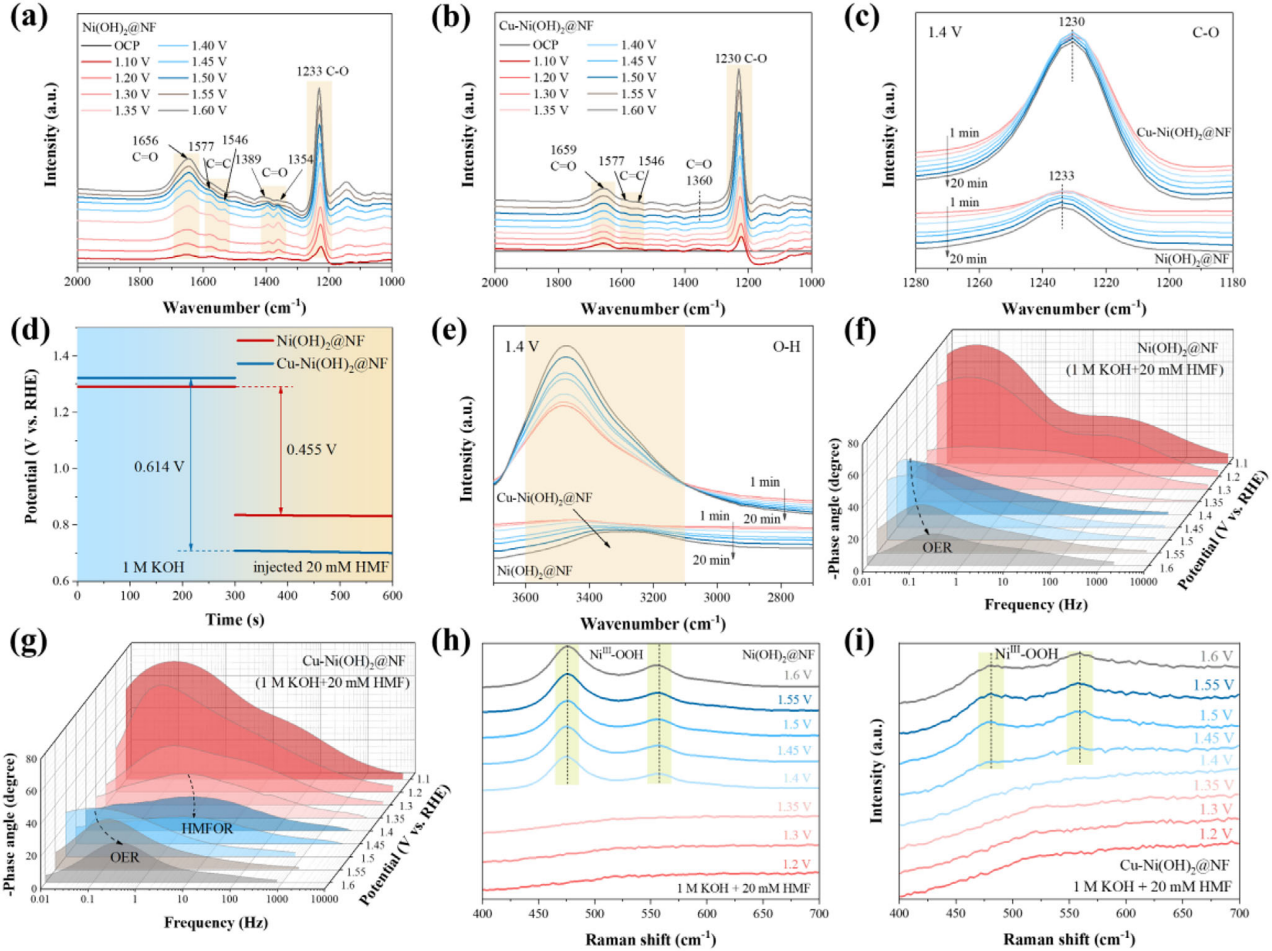
(a–c) In situ ATR‐SEIRAS spectra of (a) Ni(OH)_2_@NF; (b) Cu‐Ni(OH)_2_@NF; and (c) C─O on Ni(OH)_2_@NF and Cu‐Ni(OH)_2_@NF at 1.40 V during HMFOR. (d) OCP curves of Ni(OH)_2_@NF and Cu‐Ni(OH)_2_@NF. (e) In situ ATR‐SEIRAS spectra of OH_ads_ on Ni(OH)_2_@NF and Cu‐Ni(OH)_2_@NF at 1.40 V during HMFOR. (f,g) In situ EIS spectra of (f) Ni(OH)_2_@NF and (g) Cu‐Ni(OH)_2_@NF at different potentials in KOH with 20 mm HMF. (h,i) In situ Raman spectroscopy of (h) Ni(OH)_2_@NF and (i) Cu‐Ni(OH)_2_@NF at different potentials in KOH with 20 mm HMF.

To gain deeper insight into the catalytic reaction path, we used in situ attenuated total reflection surface‐enhanced infrared absorption spectroscopy (ATR‐SEIRAS) to monitor the adsorption of key reactants and intermediates during HMFOR (Figure S9). In Figure 4a, the peak at around 1233 cm^−1^ is identified as the C─O stretching vibration peak of the HMF molecule or intermediates [22, 43]. The characteristic peaks at 1354 and 1389 cm^−1^ belong to the symmetric vibration of O─C═O associated with the carboxylate groups in HMFCA, FFCA intermediates, and FDCA product [44]. The conjugated C═C (1546 and 1577 cm^−1^) on furan rings correspond to the generation of intermediates and FDCA products [45]. The upward peaks around 1656 cm^−1^ corresponds to the asymmetric C═O stretching vibration of HMF and FFCA intermediate [46]. Noteworthy that the intensity of peaks at 1354, 1389, 1546, and 1577 cm^−1^ first increases and then decreases with the raising of applied potential, attributing to the intermediates accumulated and consumed on the surface of Ni(OH)_2_@NF. In contrast, Cu‐Ni(OH)_2_@NF gave much weaker signals at 1360, 1546, and 1577 cm^−1^ (Figure 4b). This suppression suggests that intermediate species were more rapidly consumed, pointing to a faster conversion to FDCA.

At a fixed potential of 1.40 V vs. RHE, the C─O stretching vibration on Cu‐Ni(OH)_2_@NF is stronger than on Ni(OH)_2_@NF, and its intensity continues to grow with reaction time, which indicates that the coverage of organic reactants on Cu‐Ni(OH)_2_@NF is significantly higher (Figure 4c). In addition, the C─O peak on Cu‐Ni(OH)_2_@NF appears at a lower wavenumber than on Ni(OH)_2_@NF, indicating the stretching of the C─O bond caused by strong interactions between HMF (and its intermediates) and the catalyst surface. XPS and XAS analyses (Figure 2) reveal that Cu substitution increases the Ni valence state and shortens the Ni─O bond length, which enhances the electrophilic character of Ni sites, strengthening their interaction with the C─O bonds of organic molecules. During adsorption, the electron‐deficient high‐valence Ni sites attract electrons from the C─O bond, lowering its bond energy and extending its length. This demonstrates that Cu‐Ni(OH)_2_@NF has a stronger intrinsic adsorption ability, which facilitates the binding of HMF molecules and their intermediates throughout the reaction. To validate this effect, open‐circuit potential (OCP) tests were performed to probe the adsorption of organic molecules on the Helmholtz layer [47]. After introducing 20 mm HMF, Cu‐Ni(OH)_2_@NF had a more pronounced drop in OCP than Ni(OH)_2_@NF, confirming that Cu incorporation promotes HMF adsorption (Figure 4d). Similar OCP trends were observed for the key intermediates, including 5‐hydroxymethyl‐2‐furancarboxylic acid (HMFCA) and 2‐furancarboxylic acid (FFCA) (Figure S10), highlighting the general enhancement of organic molecule adsorption on Cu‐Ni(OH)_2_@NF.

We also examined the competitive adsorption and transformation of hydroxide ions (OH^−^). CV measurements were performed in the range 0.623–1.223 V vs. RHE (Figure S11) [44, 48]. The oxidation peak of Cu‐Ni(OH)_2_@NF is larger than that of Ni(OH)_2_@NF in both the presence and absence of HMF, illustrating a stronger intrinsic OH^−^ adsorption during the reaction. In situ ATR‐SEIRAS spectra (Figure 4e) show that Cu‐Ni(OH)_2_@NF has a higher O─H vibration peak intensity in the range of 3100–3600 cm^−1^, which indicates that Cu substitution promotes the surface coverage of adsorbed hydroxyl species (OH_ads_) [49]. Over time, the O─H peak for Ni(OH)_2_@NF gradually shifted to lower wavenumbers than those for Cu‐Ni(OH)_2_@NF, reflecting bond elongation and a lower bond energy. Such changes accelerate the deprotonation step in the competing OER, which is consistent with the performance results (Figure 3d). It is therefore reasonable to infer that Cu incorporation optimizes the intrinsic adsorption between organic reactants and OH_ads_ on the Cu‐Ni(OH)_2_@NF catalyst, while simultaneously suppressing the competing OER.

In situ electrochemical impedance spectroscopy (EIS) was carried out to deeply clarify the interfacial dynamics and electron transfer during the catalytic process. The corresponding Bode plots in the 1 m KOH electrolyte (Figure S12) show that the characteristic OER signals for Cu‐Ni(OH)_2_@NF and Ni(OH)_2_@NF start to appear in the low‐frequency region (0.01–1 Hz) since 1.40 V vs. RHE [33]. After adding HMF, Ni(OH)_2_@NF shows a similar trend of characteristic signals with and without HMF, elucidating the strong competitiveness of OER (Figure 4f; Figure S12a). In contrast, the signals attributed to HMFOR on Cu‐Ni(OH)_2_@NF appear at middle‐frequency region (1∼100 Hz) and gradually shift to higher frequencies (Figure 4g), where signals of OER are delayed to 1.50 V vs. RHE. These results verify the faster HMF oxidation rate and the inhibited OER kinetics on the surface of Cu‐Ni(OH)_2_@NF. The related equivalent circuit (Figure S13), Nyquist plots (Figure S14), and fitted parameters (Tables S3–S6) were also obtained. The larger interface impedance (R_ct_) on Cu‐Ni(OH)_2_@NF indicates an inhibited charge transfer process (Tables S3, S5, and Figure S15a), which explains the weakening of O─H cleavage during OER. Instead, during the process of HMFOR (Tables S4, S6, and Figure S15b), the R_ct_ of Cu‐Ni(OH)_2_@NF is always smaller than that of Ni(OH)_2_@NF, implying faster charge transfer on Cu‐Ni(OH)_2_@NF. Above 1.50 V vs. RHE, R_ct_ slightly increases because of the influence of the competitive OER. In conclusion, the desirable adsorption ability and conversion efficiency on Cu‐Ni(OH)_2_@NF cause faster electrocatalytic reaction kinetics [50].

In situ Raman spectroscopy was used to investigate the structural changes during the electrooxidation process. As the applied potential increased to 1.40 V vs. RHE, a pair of bending and stretching vibration signals emerged at 473 and 552 cm^−1^, corresponding to the formation of Ni^3+^‐OOH species on Cu‐Ni(OH)_2_@NF in the 1 m KOH electrolyte (Figure S16) [51]. Compared with Ni(OH)_2_@NF (Figure 4h), in the presence of HMF, the occurrence of the characteristic Ni^3+^‐OOH signals on Cu‐Ni(OH)_2_@NF is delayed to 1.45 V vs. RHE (Figure 4i), illustrating that the faster dehydrogenation of HMF consumes Ni^3+^‐OOH sites and rapidly reduces Ni^3+^ to Ni^2+^ in the voltage range 1.40–1.45 V vs. RHE.

### 2.4 Mechanism Investigation by DFT Calculations

**FIGURE 5:**
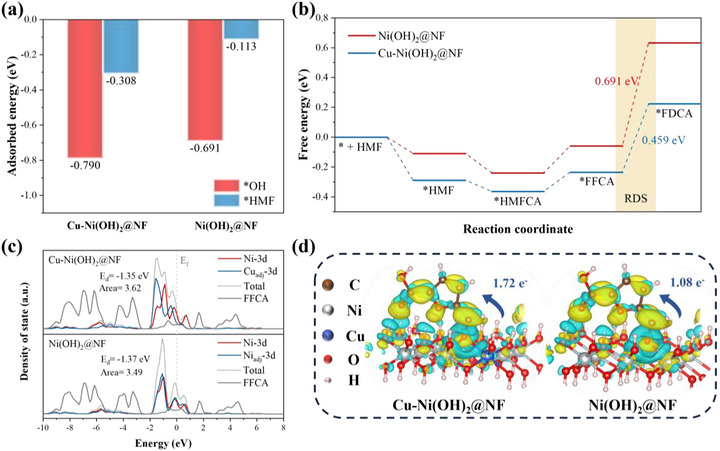
HMFOR mechanism investigation. (a) Adsorption free energy of HMF and OH_ads_ on Ni(OH)_2_@NF and Cu‐Ni(OH)_2_@NF; (b) free energy diagrams for HMFOR on the surfaces of Ni(OH)_2_@NF and Cu‐Ni(OH)_2_@NF; (c) the PDOS of the intermediate FFCA adsorptions on Ni(OH)_2_@NF and Cu‐Ni(OH)_2_@NF (Cu_adj_ and Ni_adj_ represent the substituted metals adjacent to adsorption sites); (d) Bader charge analysis of the adsorbed FFCA on Ni(OH)_2_@NF and Cu‐Ni(OH)_2_@NF models.

To fully understand the reaction mechanism of the excellent HMFOR performance on the Cu‐Ni(OH)_2_@NF catalyst, theoretical calculations were further conducted. Before that, we conducted some tests for confirming the actual active site to build more accurate structural models. We selectively poisoned the Cu sites and compared the electrochemical performance before and after toxication. LSV curves (Figure S17) show that there is no significant decrease after the deactivation of Cu sites, indicating that Ni sites act as the direct active center rather than Cu. Additionally, we conducted a pre‐oxidized test under an applied potential of 1.45 V vs. RHE for 10 min in HMF solution. The Cu 2p XPS spectrum of the pre‐oxidized Cu‐Ni(OH)_2_@NF illustrates that the oxidation state of Cu is unchanged compared to the fresh Cu‐Ni(OH)_2_@NF (Figure S18), further confirming that Cu tends to be a structural/electronic modulator rather than the direct redox‐active center for HMFOR. The optimized slab models for Ni(OH)_2_@NF and Cu‐Ni(OH)_2_@NF were developed (Figures S19 and S20). The HMF molecule preferentially chemisorbs at Ni sites adjacent to substitutional Cu (Figure S21), a local configuration that strengthens aldehyde‐end coordination and lowers the initial kinetic barrier for HMFOR. Adsorption energetics (Figure 5a) show a markedly stronger HMF (HMF_ads_) binding on Cu‐Ni(OH)_2_@NF (ΔE_HMFads_ = −0.308 eV) than on Ni(OH)_2_@NF (−0.113 eV), which is consistent with facilitated C─H activation. Meanwhile, co‐adsorbed OH^−^ (OH^−^_ads_) is stabilized (ΔE_OH_^−^_ads_ = −0.790 eV), suppressing parasitic oxygen evolution and biasing surface coverage toward HMFOR‐relevant intermediates. The full reaction coordinate (Figure 5b) identifies ^*^FFCA → ^*^FDCA as the RDS on both catalysts. Notably, the corresponding barrier for the RDS decreases from 0.691 eV on Ni(OH)_2_@NF to 0.459 eV on Cu‐Ni(OH)_2_@NF, directly enabling high‐current‐density operation. Consistently, OER analysis (Figure S22) assigns ^*^OH → ^*^O as the RDS for both surfaces, and the larger theoretical overpotential on Cu‐Ni(OH)_2_@NF (0.602 V vs. 0.480 V for Ni(OH)_2_@NF) confirms the mitigated O─H deprotonation in the presence of Cu, in line with the experimentally observed suppression of the competing OER during HMFOR.

Electronic‐structure analysis clarifies the atomic‐scale origin of these kinetics. The projected density of states (PDOS) analysis shows that the introduction of Cu shifts the Ni‐3d d‐band center upward (E_d_ = −1.35 eV vs. −1.37 eV for Ni(OH)_2_@NF), moving it closer to the Fermi level (E_f_) and increasing its affinity for the key ^*^FFCA intermediate (Figure 5c). The larger harmonic overlap between Ni‐3d states and ^*^FFCA (3.62 vs. 3.49) indicates stronger orbital coupling, while an increased total DOS near E_f_ facilitates electron extraction during the ^*^FFCA → ^*^FDCA step. Bader charge analysis (Figure 5d) indicates a larger charge transfer from the catalyst to the adsorbed FFCA on Cu‐Ni(OH)_2_@NF (+1.72 |e| vs. +1.08 |e|), populating the C═O π orbital, reducing its bond order, and thereby lowering the nucleophilic‐attack barrier of the RDS. Optimized configurations confirm the strong adsorption of FFCA on Cu‐Ni(OH)_2_@NF, promoting the sequential oxidation of adsorbed intermediates, which is the primary bottleneck step.

In parallel, Bader charge comparison across the catalysts (Figure S23) shows that Cu‐Ni(OH)_2_ transfers less charge to the adsorbed OH^−^ than its Co/Cr/Mn‐substituted analogues but more than unsubstituted Ni(OH)_2_, implying a Ni valence higher than in pure Ni(OH)_2_ yet lower than in the other three substituted systems. Consistent with their OER performance (Figure S24), Cu‐Ni(OH)_2_ has an inferior OER activity than the Co/Cr/Mn‐substituted catalysts, indicating that a moderately elevated Ni valence optimizes HMF oxidation, whereas an even higher Ni valence promotes OER and intensifies competition during HMFOR—ultimately diminishing HMF electrooxidation performance in those more OER‐active systems. Collectively, these effects optimize adsorption energetics and increase charge transfer across the key intermediates, synergistically optimizing the aldehyde C─H and O─H deprotonation of the two competing reactions to realize high‐current HMF electrooxidation.

### 2.5 Product Analysis and Integrated Electrolyzer Performance

**FIGURE 6:**
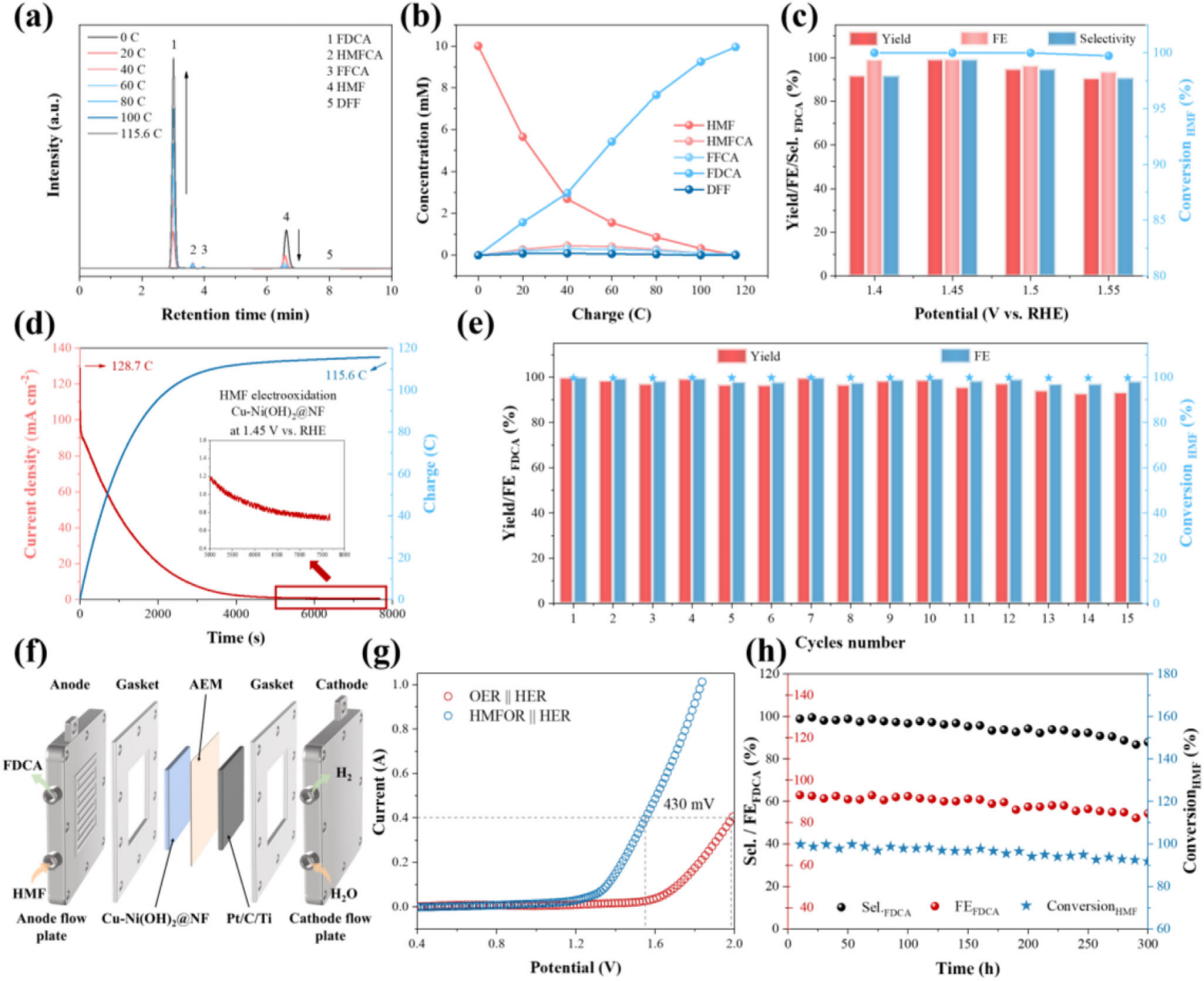
Product analysis and application studies for HMFOR. (a) HPLC chromatogram as the charge builds up at 1.45 V vs. RHE; (b) concentration changes of HMF and its oxidation products during HMFOR on Cu‐Ni(OH)_2_@NF; (c) FE_FDCA_, yield_FDCA_, selectivity_FDCA_ and HMF conversion at different potentials in the 10 mm HMF solution; (d) i‐Q‐t curves of HMF oxidation at 1.45 V vs. RHE; (e) FE_FDCA_, yield_FDCA_ and HMF conversion after 15 cycles at 1.45 V vs. RHE on Cu‐Ni(OH)_2_@NF; (f) schematic diagram of the MEA electrolyzer for the coupled HMFOR || HER system (AEM: anion exchange membrane); (g) LSV curves of Cu‐Ni(OH)_2_@NF in the MEA electrolyzer at room temperature; (h) stability evaluation for the MEA at 1.65 V.

Encouraged by the promising electrocatalytic activities of Cu‐Ni(OH)_2_@NF, we collected and detected the products during HMFOR to evaluate the conversion efficiency. The chronoamperometry electrolysis was carried out in a divided H‐type cell containing 20 mL 1 m KOH+10 mm HMF solution, and the collected electrolyte was tested by high‐performance liquid chromatography (HPLC) to determine the components and their concentrations. At an applied potential of 1.45 V vs. RHE, the intensity of the HMF signal gradually decreases with the increase of passed charge, accompanied by an increase of the FDCA signal (Figure 6a), indicating the gradual conversion of HMF to FDCA. Based on the standard curves of different organic reactants and intermediates (Figure S25), the quantified concentration curves show that only a few of HMFCA and FFCA are traced in the electrocatalytic process, while the concentration of 2,5‐diformylfuran (DFF) is almost undetectable (Figure 6b). These results provide further experimental validation that the HMF electrooxidation follows the pathway HMF→HMFCA→FFCA→FDCA (Figure S26) [52, 53], in agreement with the proposed mechanism.

We also tested the HMFOR performance at potentials ranging from 1.40 to 1.55 V vs. RHE, and calculated the conversion of HMF, yield, FE, and selectivity of FDCA (Figure 6c). Compared with Ni(OH)_2_@NF (Figure S27), the Cu‐Ni(OH)_2_@NF catalyst achieves a high HMF conversion that is close to 100%, the yield_FDCA_ reaches to 99.3%, and the FE_FDCA_ is as high as 99.5% under an applied potential of 1.45 V vs. RHE, implying that Cu substitution plays a dominant role in the high‐performance HMFOR. Besides, with the increase of passed charge, there is no distinct plateau in the i‐t curve even at a low substrate concentration, confirming the continuity of HMFOR on Cu‐Ni(OH)_2_@NF (Figure 6d).

Based on the above conclusions, the optimal potential for HMFOR is 1.45 V vs. RHE for the Cu‐Ni(OH)_2_@NF catalyst. We therefore performed the reaction under this condition for 15 cycles (Figure S28). From Figure 6e, we see that the performance remains almost unchanged over 15 continuous cycles, with an HMF conversion > 99.6%, a yield_FDCA_ > 92.9%, and a FE_FDCA_ > 97.1%, which suggests the superior stability of Cu‐Ni(OH)_2_@NF in long‐term electrooxidation tests. Moreover, after long‐term cycling experiments, SEM and EDS images of the recovered catalyst show that there is no obvious change in the morphology of the material (Figure S29), which indicates the outstanding structural stability of Cu‐Ni(OH)_2_@NF. The XRD pattern of Cu‐Ni(OH)_2_@NF also confirms the retention of the initial crystalline phase (Figure S30). In XPS spectra (Figure S31), the electronic structure and local coordination environment of Ni and Cu are essentially preserved, indicating the stable chemical state of the catalyst under high current densities. The ICP‐OES result after the reaction (Table S7) indicates the trace dissolution of Ni and Cu, which can be attributed to the removal of a small fraction of unstable surface sites during the initial stabilization period. After this minor leaching, the remaining catalyst achieves a stable state.

We designed a 2 × 2 cm^2^ membrane electrode assembly (MEA) flow electrolyzer for the integrating of HMFOR and HER (Figure 6f) to assess the activity of the catalyst in close to practical situations [54]. In the circulated homogeneous electrolyte (1 m KOH with 100 mm HMF), Cu‐Ni(OH)_2_@NF and Pt/C loaded on Ti (Pt/C/Ti) were used as anode and cathode electrocatalysts, respectively. For comparison, the OER||HER system equipped with the same anode and cathode was evaluated in 1.0 m KOH without HMF under identical conditions. LSV curves reveal the superior efficiency of the HMFOR || HER system, and up to 1 A can be realized at a low cell voltage of 1.82 V (Figure 6g). To achieve a current of 400 mA, the cell voltage of HMFOR || HER system is approximately 430 mV lower than that of OER || HER system, reducing the energy input. At a constant applied potential of 1.65 V, our HMFOR || HER system achieves excellent stability for nearly 300 h, with negligible decrease of the selectively_FDCA_, FE_FDCA_, and HMF conversion (Figure 6h). After electrolysis, we collected the FDCA product by acidification and filtration. HPLC, XRD, and FT‐IR results verify the production of high‐purity FDCA, matching well with the standard FDCA (Figures S32–S34). Coupling HMFOR with HER in our MEA device drastically reduces the energy consumption for H_2_ production while simultaneously generating high‐value FDCA with high selectivity and stability, demonstrating a promising alternative to traditional electrolysis.

## 3 Conclusions

The precise valence modulation of Ni sites in a Cu‐substituted Ni(OH)_2_@NF catalyst, simultaneously accelerates the aldehyde oxidation and suppresses the parasitic OER to achieve a high current density. A combination of theoretical calculations and experimental results indicates that the incorporation of Cu changes the local electronic structure of Ni sites, creating the unique orthogonal activity that selectively activating the aldehyde C─H during HMFOR while remaining inert toward the O─H cleavage in OER. Consequently, the Cu‐Ni(OH)_2_@NF catalyst achieves an ampere‐level current density of 1000 mA cm^−2^ at 1.55 V vs. RHE and 1200 mA cm^−2^ at 1.60 V vs. RHE in 1 m KOH with 50 mm HMF. Remarkably, the key reaction barrier of the RDS of aldehyde‐to‐acid conversion is significantly reduced on Cu‐Ni(OH)_2_@NF, realizing further oxidation with an impressively high FE_FDCA_ of 99.5%. The stability over 15 continuous cycles confirms the application potential of Cu‐Ni(OH)_2_@NF as a catalyst for long‐term electrooxidation. The orthogonal design established in this work, which selectively optimizes the deprotonation kinetics of the two competing reactions, paves the way for the efficient and sustainable electrochemical production of value‐added chemicals from biomass.

## Conflicts of Interest

The authors declare no conflicts of interest.

## Supporting information




**Supporting File**: advs75458‐sup‐0001‐SuppMat.pdf.

## Data Availability

The data that support the findings of this study are available from the corresponding author upon reasonable request.
