# Supplementary material for: Orthogonal Design of Competing Deprotonation Process in Cation‐Mediated Ni(OH)2 for Achieving Industrial Level Biomass Electrooxidation
Source: Adv Sci (Weinh). 2026 Apr 27;13(41):e75458. doi: 10.1002/advs.75458 (PMC13335460; doi:10.1002/advs.75458)
Supplement: Supplementary file 1 — Supporting File: advs75458‐sup‐0001‐SuppMat.pdf. [file ADVS-13-e75458-s001.pdf]

Supplementary Information

Orthogonal Design of Competing Deprotonation Process in Cation-Mediated $Ni(OH)_2$ for Achieving Industrial Level Biomass Electrooxidation

Junge Yang[#], Zhengjie Chen[#], Lili Zhang, Tao Zhang, Shida Bao, Jing Peng[*], Hui Pan[*], Hui-Ming Cheng[*]

Equal major contributors

* Corresponding author

Jing Peng: jing.peng@siat.ac.cn (email)

Hui Pan: huipan@um.edu.mo (email)

Hui-Ming Cheng: hm.cheng@siat.ac.cn (email)

Experimental Section

Chemicals:

Nickel foam (NF) was purchased from Suzhou Sinero Technology Company. Acetone, hydrochloric acid (HCl) and ethanol (EtOH) were purchased from Beijing Chemical Reagents Company. Nickel nitrate hexahydrate ($Ni(NO_3)_2 \cdot 6H_2O$), urea ($CO(NH_2)_2$), cupric chloride dihydrate ($CuCl_2 \cdot 2H_2O$), chromic chloride hexahydrate ($CrCl_3 \cdot 6H_2O$), manganese chloride tetrahydrate ($MnCl_2 \cdot 4H_2O$), cobalt chloride hexahydrate ($CoCl_2 \cdot 6H_2O$), potassium hydroxide (KOH), 5-Hydroxymethylfurfural (HMF), 2,5-diformylfuran (DFF), 5-hydroxymethyl-2-furancarboxylic acid (HMFCA), 5-formyl-2-furancarboxylic acid (FFCA), 2,5-furandicarboxylic acid (FDCA) and Dimethyl sulfoxide (DMSO) were provided by Shanghai Aladdin Reagent Co., Ltd. All the reagents are of analytical purity and used without further purification. Deionized water (DI water) (18.2 MΩ · cm) used in all experiments was prepared by passing through an ultra-pure purification system.

Synthesis of $Ni(OH)_2$@NF:

Prior to use, the NF (3 cm × 4 cm) was pre-treated by ultrasonic treatment for 25 min each in acetone, 3 M HCl and DI water,. A typical hydrothermal method was used for the synthesis of nickel hydroxide grown on nickel foam. The clean NF was vertically placed in 60 mL of a mixture of $Ni(NO_3)_2 \cdot H_2O$ (3 mmol) and $CO(NH_2)_2$ (6 mmol), then transferred into a 100 mL Teflon-lined autoclave, and kept at 200 ° C for 5 h. Subsequently, the product was washed with DI water and ethanol for several times and dried for 5 h in the vacuum at 60 ° C.

Synthesis of Cu-$Ni(OH)_2$@NF catalyst:

30 mL 3 mM $CuCl_2 \cdot 2H_2O$ solution was transferred into a 50 mL Teflon autoclave, and perpendicularly placed a piece of $Ni(OH)_2$@NF (2 cm × 3 cm). After sealing, the autoclave was heated to 140 ° C for 4 h. The final product was washed with DI water and ethanol several times and dried for 5 h in the vacuum at 60 ° C.

Synthesis of other catalysts M-$Ni(OH)_2$@NF:

The preparation of the other catalysts M-$Ni(OH)_2$@NF were the same as that of Cu-$Ni(OH)_2$@NF except that $CuCl_2 \cdot 2H_2O$ was replaced by $CrCl_3 \cdot 6H_2O$, $MnCl_2 \cdot 4H_2O$ and $CoCl_2.6H_2O$, respectively.

Material characterization：

The morphology of the electrocatalysts were characterized by a Zeiss scanning electron

microscope (SEM) and a Talos F200S high-resolution transmission electron microscope (HR-TEM). The mass loading of Ni, O and Cu in Cu-$Ni(OH)_2$@NF was measured by inductively coupled plasma optical emission spectrometry (ICP-OES). X-ray diffraction (XRD) patterns were collected using a Rigaku SmartLab diffractometer equipped with Cu Kα radiation (45 kV, 200 mA, λ=1.5418 Å) for 2θ angles from 10 ° to 80 ° at a speed of 6 ° min^{-1}. The chemical composition and valence states were investigated using a X-ray photoelectron spectrometer (XPS, PHI 5000 VersaProbe III, Al Kα X-ray, hν = 1486.7 eV, using C 1 s, 284.8 eV for calibration). XAFS measurements at the Ni k-edge in both transmission (for Ni foil) and fluorescence (for catalysts) modes were acquired at the Shanghai Synchrotron Radiation Facility and the data were collected with a double-crystal Si (111) monochromator. All the energy were calibrated according to the absorption edge of Ni foil. The raw data analysis was performed by using Athena, Artemis, and hama software.

Electrochemical measurements:

All the electrochemical performance tests were conducted in a three-electrode divided H-type electrochemical cell system (at room temperature of 25 °C) on an Corrtest (CS310X) electrochemical workstation. The electrocatalysts were used as the working electrode, Hg/HgO was taken as the reference electrode (RE), and platinum (Pt) (1 cm × 1 cm) was used as the counter electrode (CE). The anode and cathode were separated by a Nafion 117 exchange membrane, and filled with 1 M KOH + 20 mM HMF and 1 M KOH solution as anolyte and catholyte, respectively. The electrochemical tests were carried out under continuous stirring at 1000 rmp, and with 90 % iR compensation. All the potentials were converted to a reversible hydrogen electrode (RHE) using the following equation:

$$E_{RHE} = E_{measured} + E_{Hg/HgO} + 0.0591 \times pH$$

Linear sweep voltammetry (LSV) measurements were conducted at a scan rate of 5 $mV \cdot s^{-1}$. The electrochemically active surface area (ECSA) was estimated from the electrical double-layer capacitances (C_{dl}), which were derived from cyclic voltammetry (CV) in the potential range of 0.15 V ~ 0.25 V vs. Hg/HgO under different scan rates (20 ~ 120 mV s^{-1}). Open-circuit potential (OCP) tests were recorded in 1 M KOH with and without HMF. Electrochemical impedance spectroscopy (EIS) tests were performed in the potential range 1.1 V ~ 1.6 V vs. RHE with a frequency range of 0.01 Hz ~ 100 kHz and an amplitude of 10 mV. The stability evaluation was investigated by chronoamperometry (i-t curves at a potential of 1.45 V).

In-situ infrared (IR) spectroscopy test:

Attenuated total reflection surface-enhanced infrared absorption spectroscopy (ATR-SEIRAS) was run using a Nicolet iS50 FTIR spectrometer equipped with a narrow band MCT-A detector and an in-situ IR optical accessory (SPEC-I, Shanghai Yuanfang Tech.)

at an incidence angle of ca. 60°. The IR spectra were collected with unpolarized IR radiation at a spectral resolution of 8 cm^{-1}. All spectra were shown in absorbance, defined as $-\log(R/R_0)$, where R and R_0 represent the sample and reference single-beam spectra, respectively. A CHI 760 electrochemistry workstation (CH Instruments, Inc.) was used for potential control and current measurements, and Pt mesh and Hg/HgO were used as the CE and the RE, respectively.

In-situ Raman spectroscopy test:

A Raman spectrometer (XploRA PLUS HORIBA) was used to record the corresponding Raman spectra of oxidizing $Ni(OH)_2$@NF and Cu-$Ni(OH)_2$@NF at different potentials. The excitation laser power was 10 mW and the line used was 638 nm. The electrochemical signal was input through CHI 760E, and Hg/HgO and Pt wire were used as the RE and CE in a custom-made in-situ Raman cell. Before collecting the spectra, all the electrocatalysts were pre-oxidized in the electrolyte (1 M KOH with and without HMF) by chronoamperometry for 200 s.

Product analysis:

The anolyte HMF and its oxidation products (HMFCA, FFCA, FDCA and DFF) were collected and determined by a high performance liquid chromatograph instrument (HPLC, SHIMADZU LC-20A) with a diode array detector set at 254 nm, and equipped with a 4.6 mm × 250 mm Shim-pack GIST 5-μm C18 column. The HPLC eluent consisted of 5 mM ammonium formate (FA) solution and methanol (7:3) with a flow rate of 1.5 mL min^{-1}. After reaction, the anolyte was diluted to 10 times the volume using DI water, and 10 μL of this solution was removed for detecting the concentrations. For the stability test, the volume of the anolyte was 20 mL with 10 mM HMF. The theoretical charge of the HMF oxidation reaction was calculated using the following formula:

$$6 \times (1.6\times10^{-19}\ C) \times 0.02\ L \times (0.01\ mol\ L^{-1}) \times (6.02\times10^{23}\ mol\ L^{-1}) = 115.6\ C$$

The HMF conversion, FDCA yield, faradaic efficiency (FE) and selectivity were calculated using the following equations:

$$\text{HMF conversion (\%)} = (n_{\text{consumed HMF}}/n_{\text{initial HMF}}) \times 100\ \%$$

$$\text{FDCA yield (\%)} = (n_{\text{generated FDCA}}/n_{\text{initial HMF}}) \times 100\ \%$$

$$\text{FE (\%)} = [n_{\text{generated FDCA}}/(\text{Charge}/(6\times F))] \times 100\ \%$$

$$\text{FDCA selectivity (\%)} = (n_{\text{generated FDCA}}/\ n_{\text{consumed HMF}}) \times 100\ \%$$

where n is the molar number of the reactant, calculated from HPLC data, and F is the Faraday constant (96485 C mol^{-1}).

Computational Methodology:

First-principles calculations based on density functional theory (DFT) were carried out using the Vienna Ab initio Simulation Package (VASP).[1] The exchange–correlation interactions were described within the generalized gradient approximation (GGA) using the Perdew–Burke–Ernzerhof (PBE) functional.[2] A plane-wave basis set with a cutoff energy of 400 eV was used to ensure the convergence of the total energies. The Brillouin zone was sampled with a Gamma-centered k-point grid of dimensions 3×3×1, which was chosen to strike a balance between computational efficiency and accuracy. Electronic relaxation was considered to have converged when the energy change between successive steps was less than 10^{-5} eV, and ionic relaxations were continued until the maximum force on any atom was below 0.05 eV/Å. To avoid artificial interactions between periodic images, a vacuum layer of 20 Å was inserted along the non-periodic direction (e.g., the z-axis in surface models).

The Gibbs free energy change (ΔG) under standard conditions was calculated using the thermodynamic relation:

$$\Delta G = \Delta E + \Delta ZPE - T\Delta S$$

where ΔE is the reaction energy difference obtained from the DFT total energies. ΔZPE is the change in zero-point energy, which was derived from vibrational frequency calculations performed via the finite-displacement method using the Phonopy package.[3] The entropy term TΔS was evaluated from the vibrational density of states at 298.15 K, following well-established practices for solid-state materials.[4]

Product purification:

The FDCA product was isolated from the post-electrolysis mixture by acid precipitation. The anolyte was collected and cooled in an ice bath, and HCl was added dropwise under stirring until pH 2.5-3.0. The resulting precipitate was collected by vacuum filtration, washed with ice-cold water, and dried to obtain crude FDCA. Purification was achieved via the recrystallization from a DMSO/water mixture. The crude solid was dissolved in a minimal volume of hot DMSO. Hot deionized water was added dropwise to the stirred solution until cloudiness persisted. The mixture was cooled to room temperature and then placed in an ice bath to complete crystallization. The purified crystals were isolated by filtration, washed with a cold 1:1 (v/v) water/DMSO mixture followed by ice-cold water, and dried under vacuum.

[1] Kresse, G.; Furthmüller, J. Efficient Iterative Schemes for Ab Initio Total-Energy Calculations Using a Plane-Wave Basis Set. Phys. Rev. B 1996, 54, 11169–11186.

[2] Perdew, J. P.; Burke, K.; Ernzerhof, M. Generalized Gradient Approximation Made Simple. Phys. Rev. Lett. 1996, 77, 3865–3868.

[3] Togo, A.; Tanaka, I. First Principles Phonon Calculations in Materials Science. Scr. Mater. 2015,108, 1–5.

[4] Fultz, B. Vibrational Thermodynamics of Materials. Prog. Mater. Sci. 2010,55, 247–352.

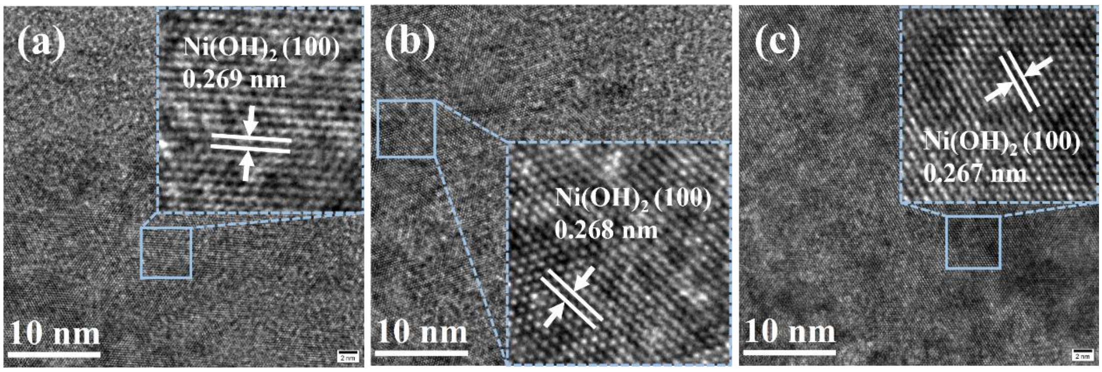

Figure S1. HRTEM images of (a)Cr-$Ni(OH)_2$@NF; (b) Mn-$Ni(OH)_2$@NF; and (c) Co-$Ni(OH)_2$@NF.

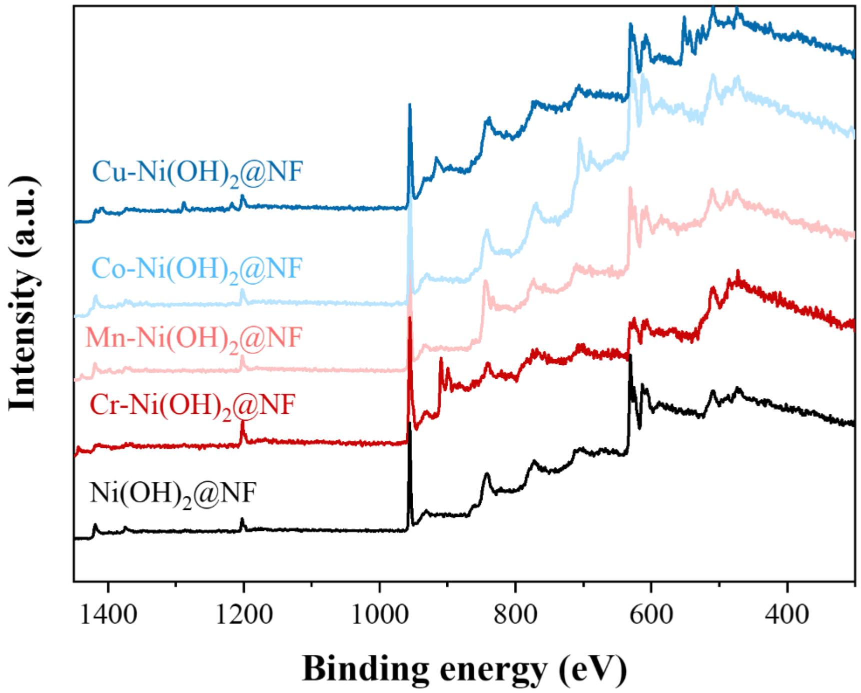

Figure S2. The XPS spectra of $Ni(OH)_2$@NF and M-$Ni(OH)_2$@NF (M = Cr, Mn, Co, Cu).

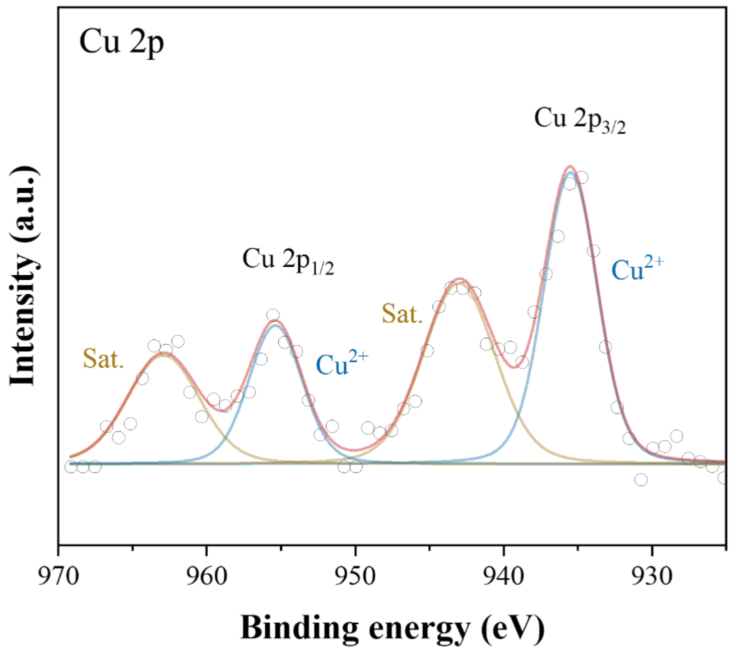

Figure S3. High-resolution XPS spectra of Cu 2p in Cu-$Ni(OH)_2$@NF.

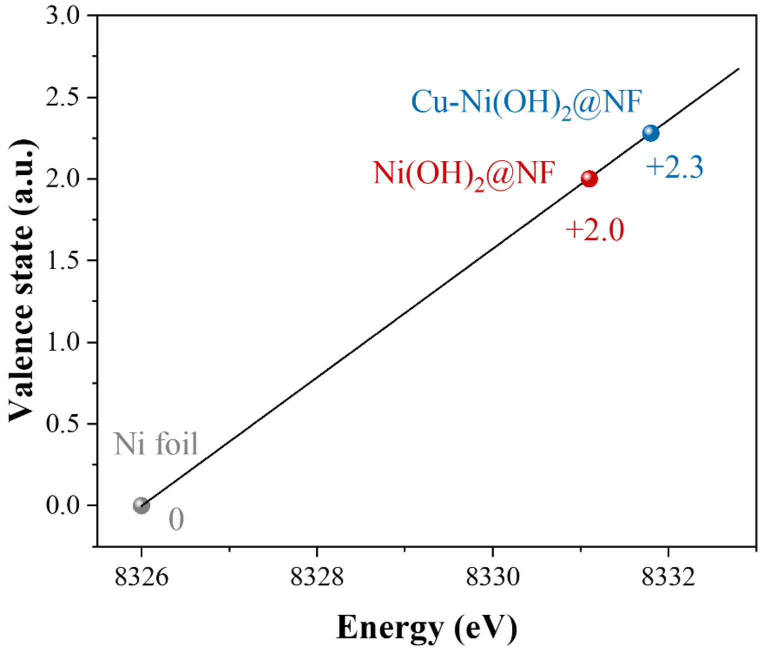

Figure S4. The average oxidation state of Ni as a function of the Ni K-edge energy.

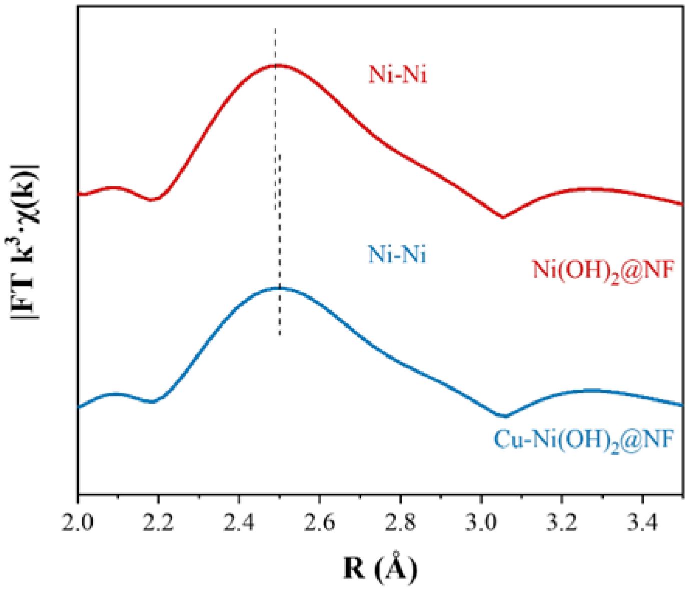

Figure S5. FT-EXAFS spectra of the Ni K-edge.

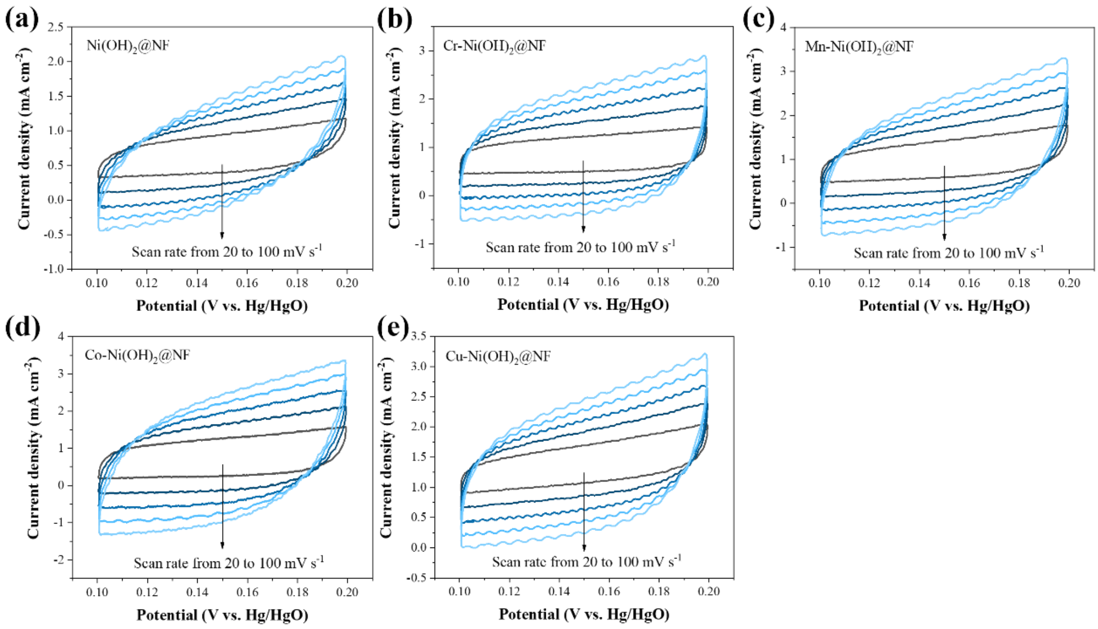

Figure S6. CV curves of (a) $Ni(OH)_2$@NF; (b) Cr-$Ni(OH)_2$@NF; (c) Mn-$Ni(OH)_2$@NF; (d) Co-$Ni(OH)_2$@NF; and (e) Cu-$Ni(OH)_2$@NF at different scan rates in 1 M KOH with 20 mM HMF.

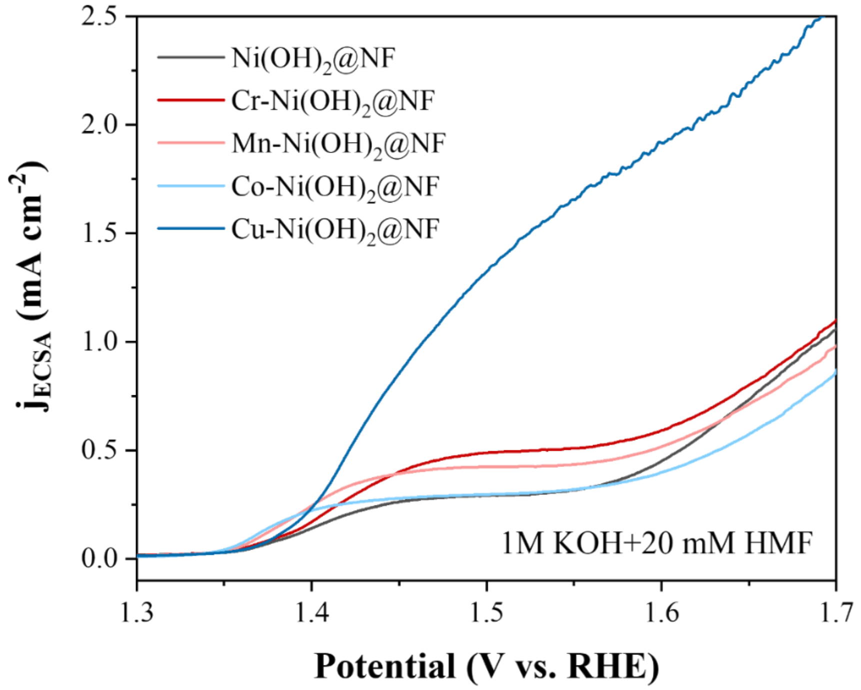

Figure S7. LSV polarization curves normalized by ECSA.

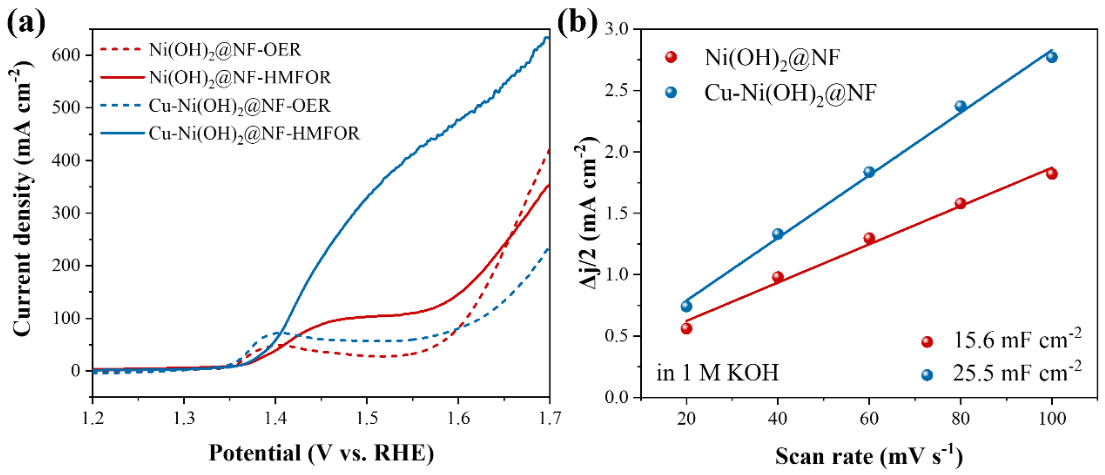

Figure S8. (a) LSV polarization curves of $Ni(OH)_2$@NF and Cu-$Ni(OH)_2$@NF in 1 M KOH with and without 20 mM HMF; (b) C_{dl} values of $Ni(OH)_2$@NF and Cu-$Ni(OH)_2$@NF in 1 M KOH.

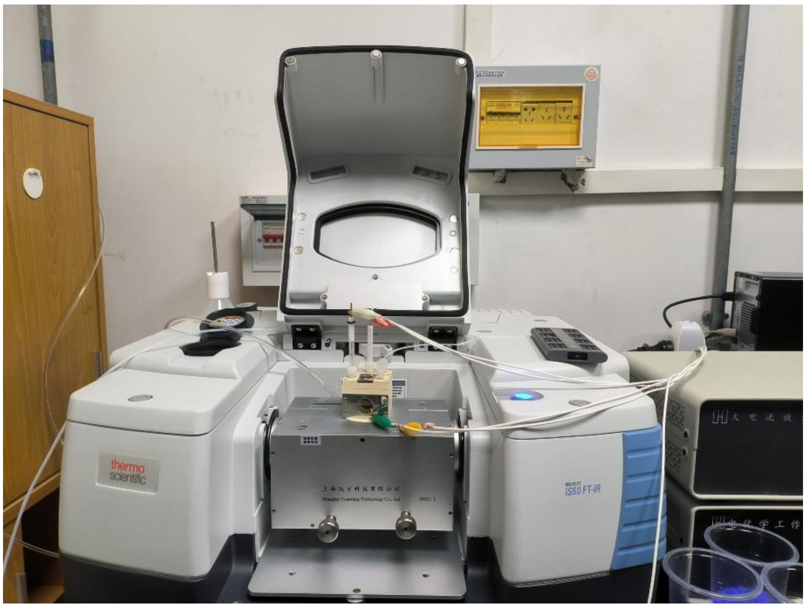

Figure S9. Photograph of the in-situ ATR-SEIRAS instrument during electrochemical measurements.

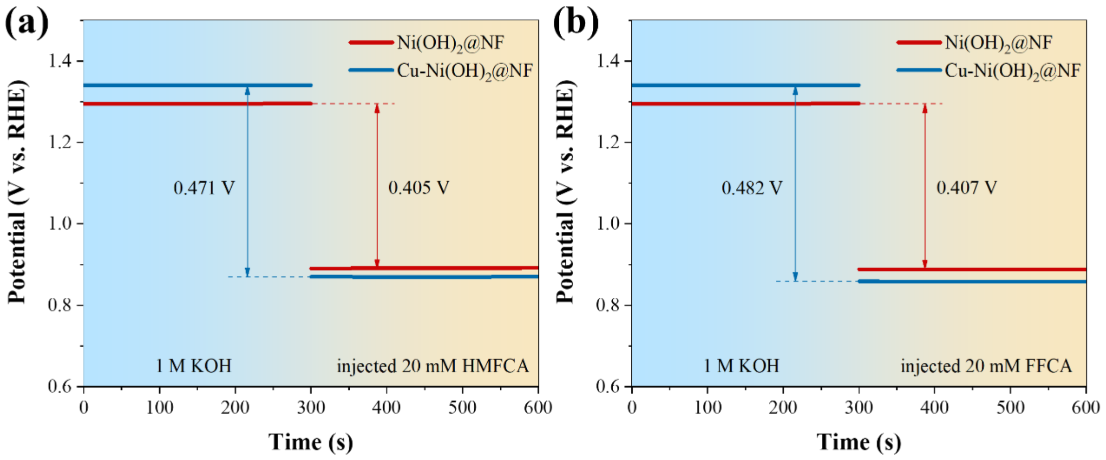

Figure S10. OCP curves of $Ni(OH)_2$@NF and Cu-$Ni(OH)_2$@NF in an alkaline electrolyte with (a) 20 mM HMFCA and (b) 20 mM FFCA.

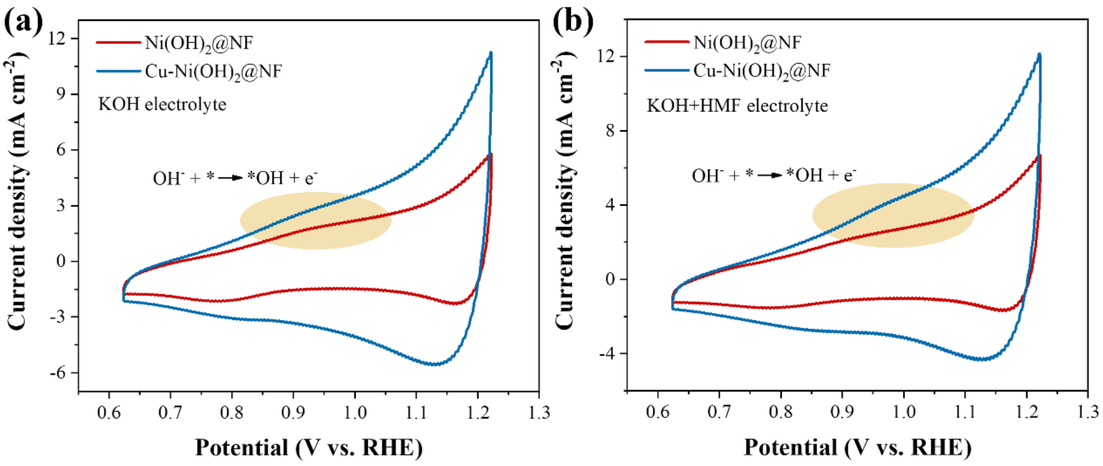

Figure S11. CV curves of $Ni(OH)_2$@NF and Cu-$Ni(OH)_2$@NF within a low potential range in 1 M KOH (a) without and (b) with HMF.

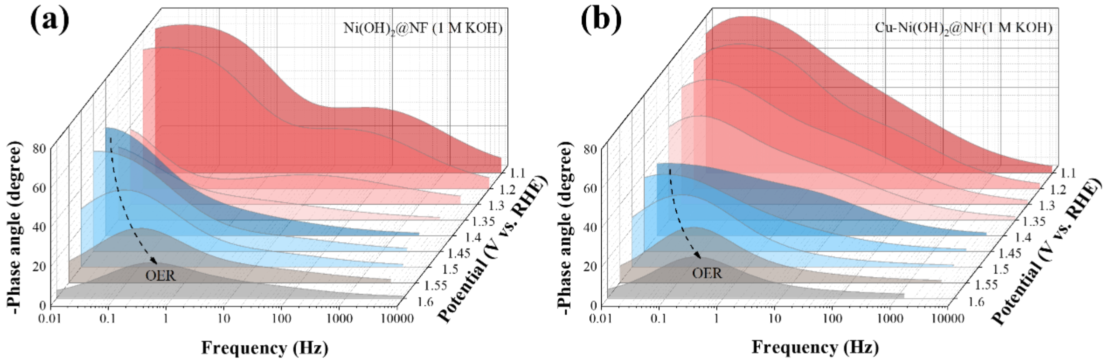

Figure S12. In-situ EIS spectra of (a) $Ni(OH)_2$@NF and (b) Cu-$Ni(OH)_2$@NF at different potentials in 1 M KOH.

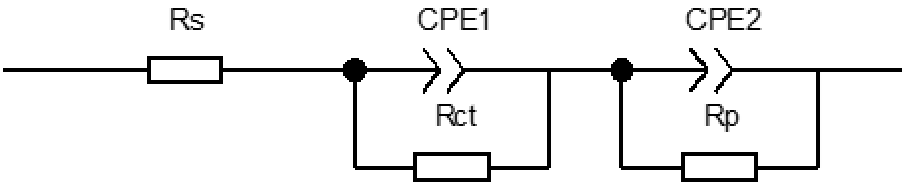

Figure S13. The simulated equivalent circuit during the reaction.

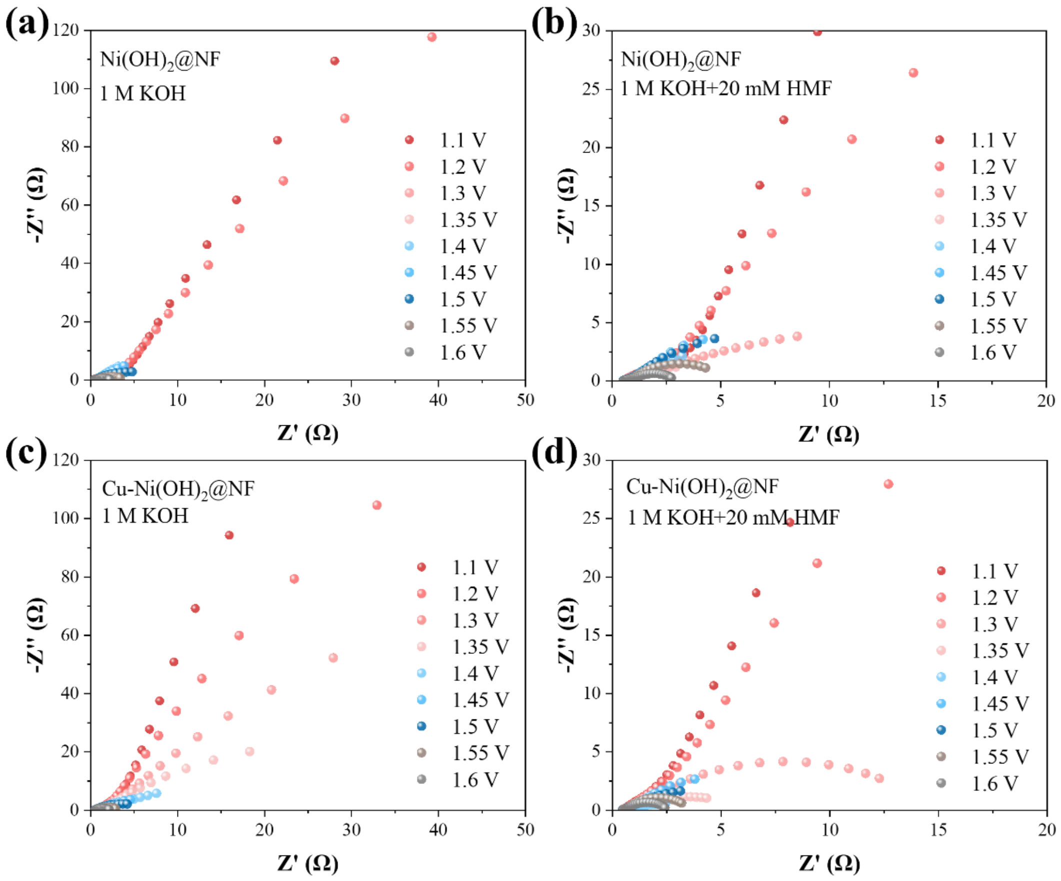

Figure S14. Nyquist plots of $Ni(OH)_2$@NF and Cu-$Ni(OH)_2$@NF in KOH (a, c) without and (b, d) with HMF.

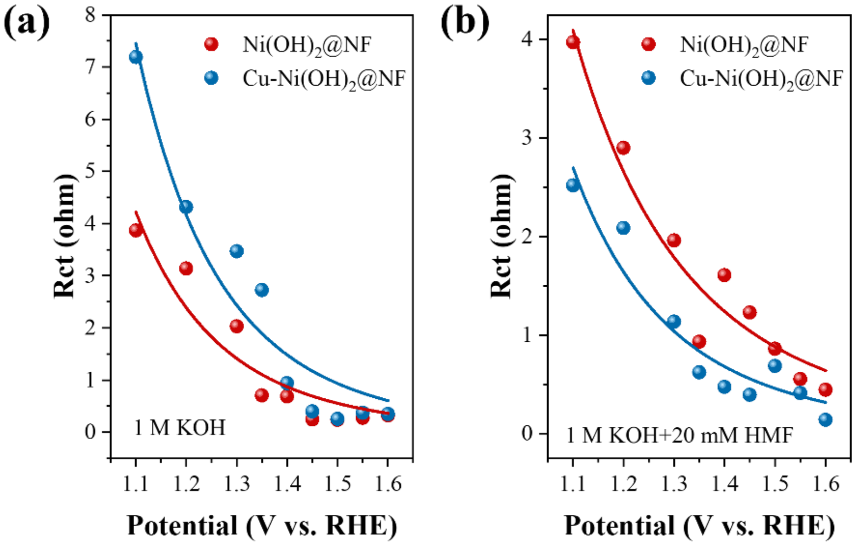

Figure S15. Fitting R_{ct} values of $Ni(OH)_2$@NF and Cu-$Ni(OH)_2$@NF during (a) OER and (b) HMFOR process.

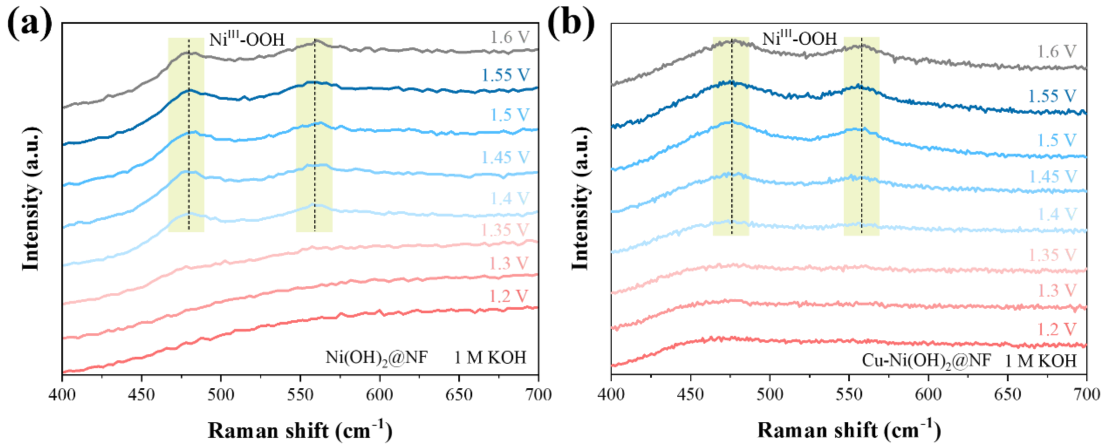

Figure S16. In-situ Raman spectroscopy of (a) $Ni(OH)_2$@NF and (b) Cu-$Ni(OH)_2$@NF at different potentials in 1 M KOH.

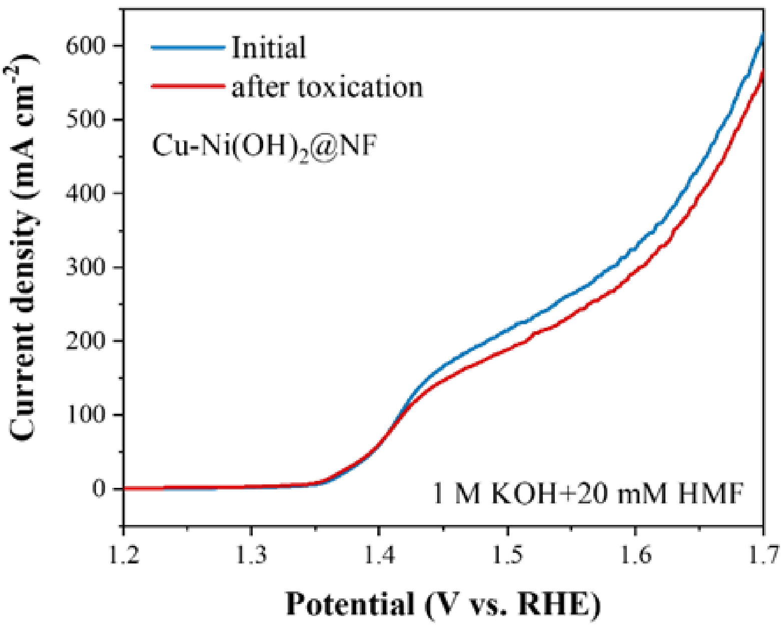

Figure S17. LSV curves of Cu-Ni$(OH)_2$@NF before and after Cu site toxication.

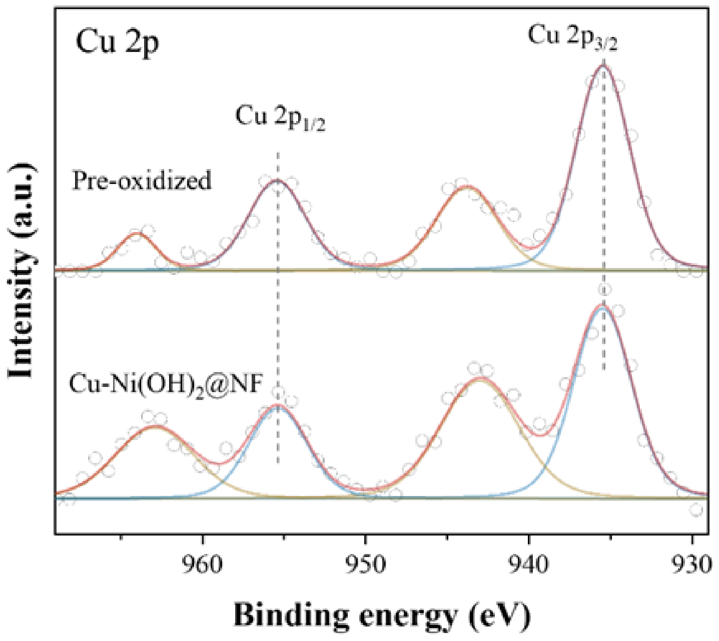

Figure S18. Cu 2p XPS spectra in Cu-Ni(OH)$_2$@NF before and after pre-oxidized test.

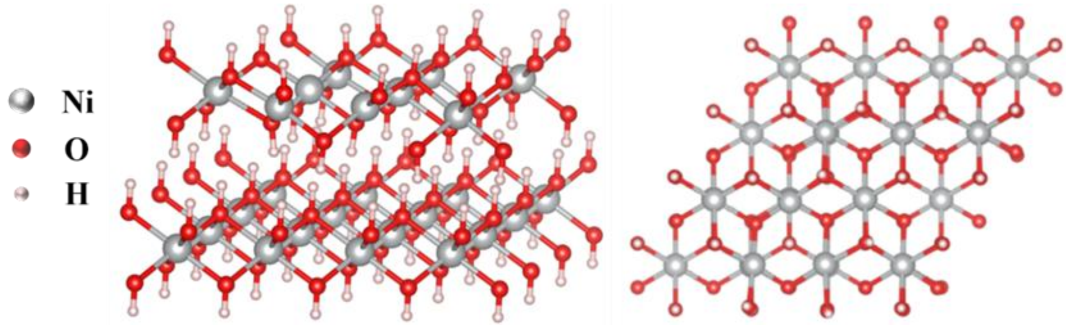

Figure S19. The side and top view of optimized model of $Ni(OH)_2$@NF.

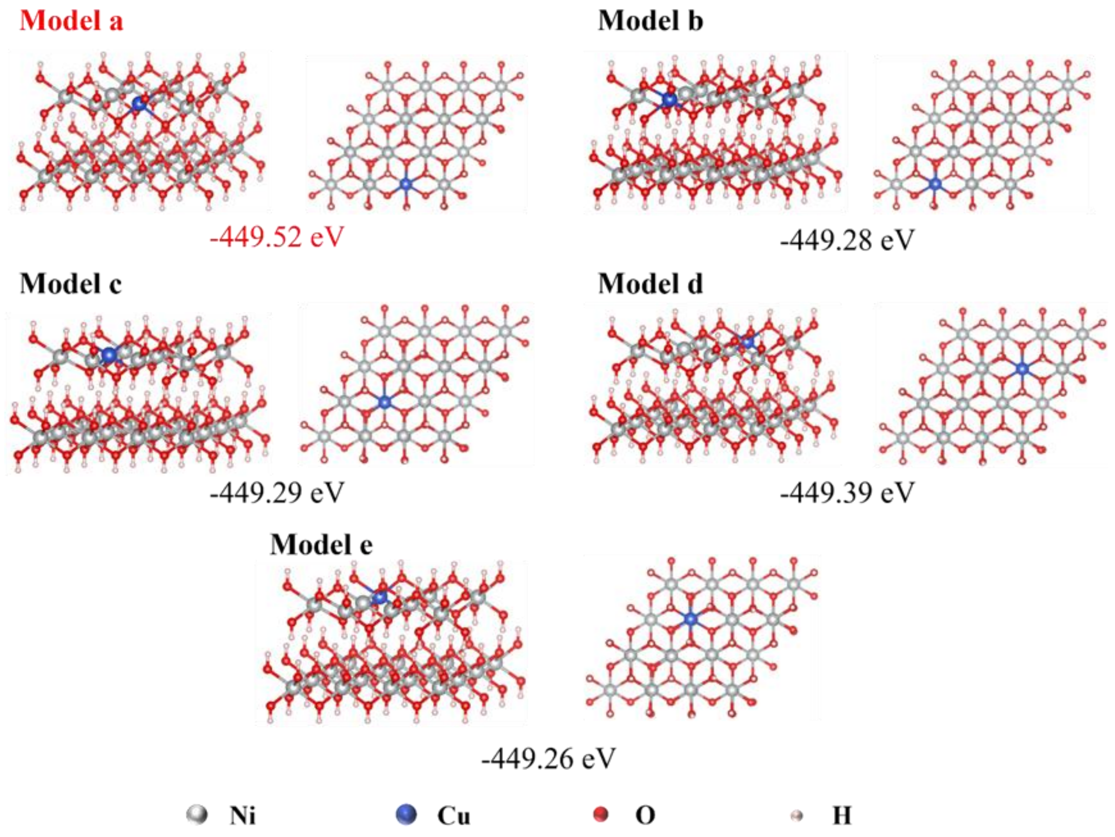

Figure S20. The side and top view of optimized model of Cu-Ni$(OH)_2$@NF (with different substitution sites).

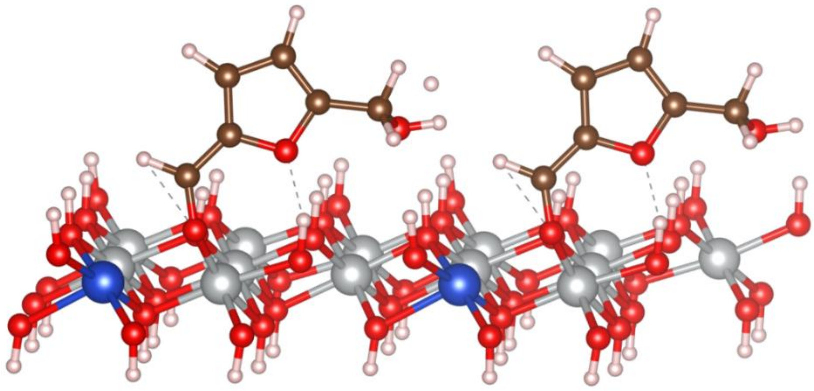

Figure S21. Adsorption configuration model of HMF on Cu-Ni$(OH)_2$@NF catalyst.

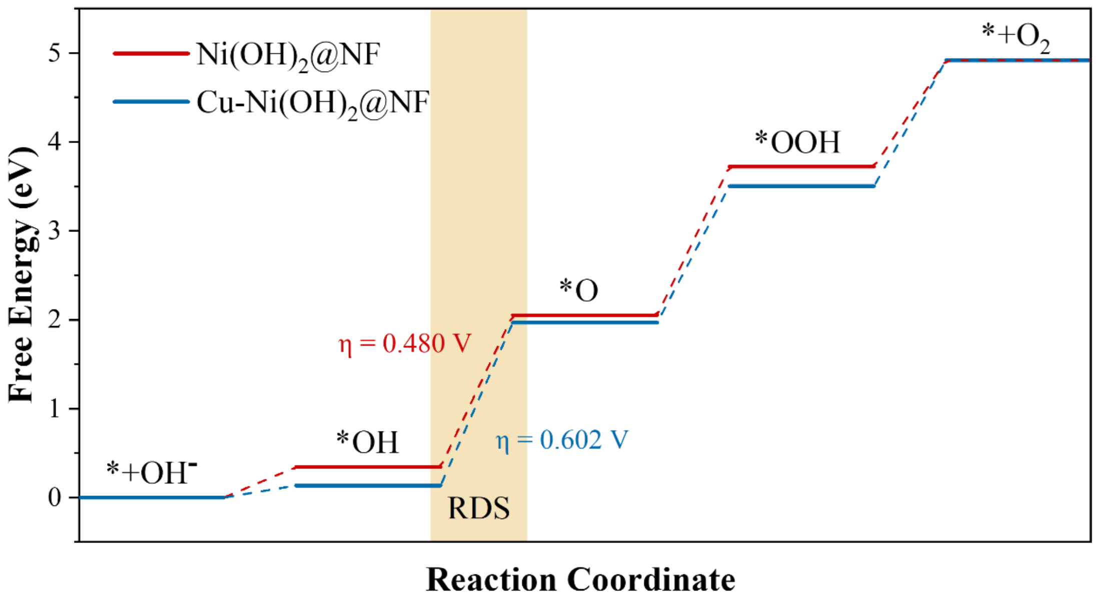

Figure S22. Free energy diagrams for OER on the surfaces of $Ni(OH)_2$@NF and Cu-$Ni(OH)_2$@NF.

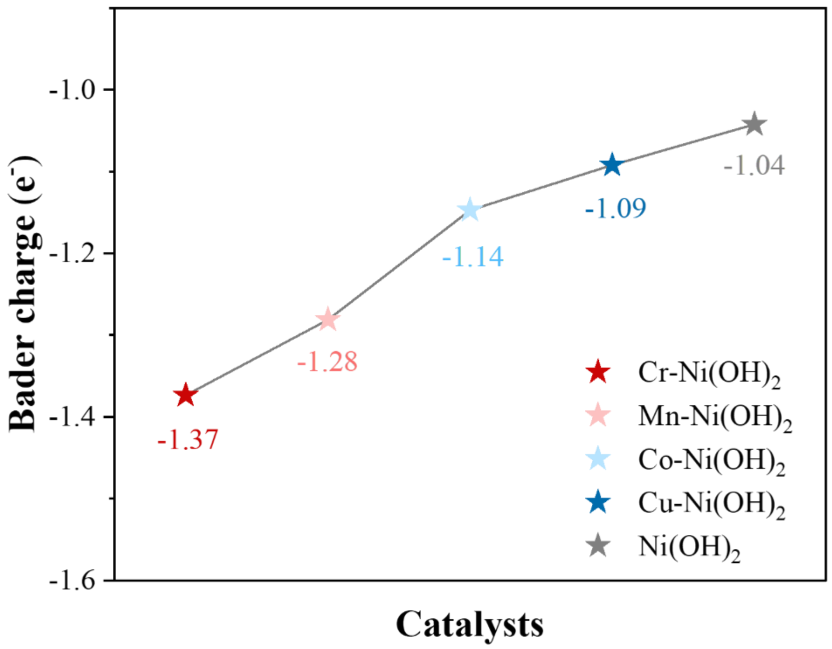

Figure S23. Bader charge analysis of the adsorbed OH^- on M-$Ni(OH)_2$@NF and $Ni(OH)_2$@NF models.

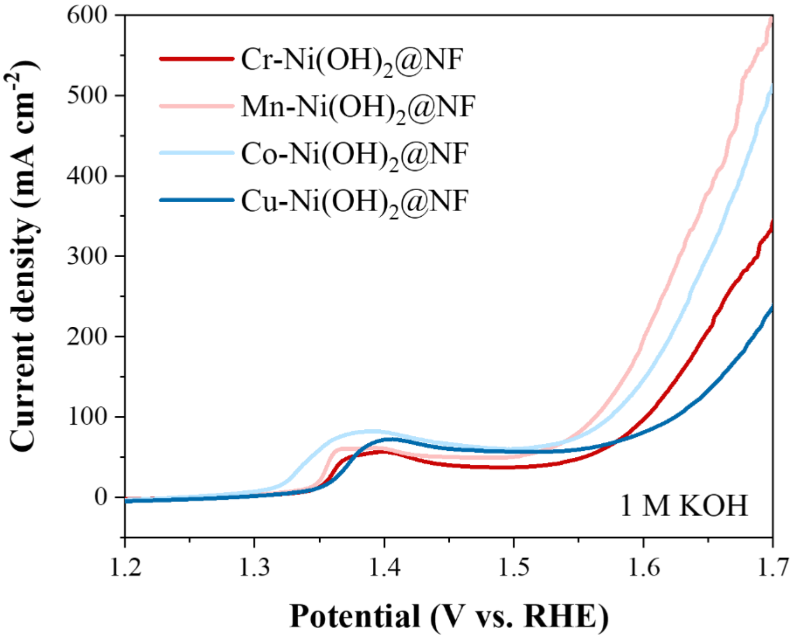

Figure S24. LSV polarization curves of M-Ni(OH)$_2$@NF in 1 M KOH.

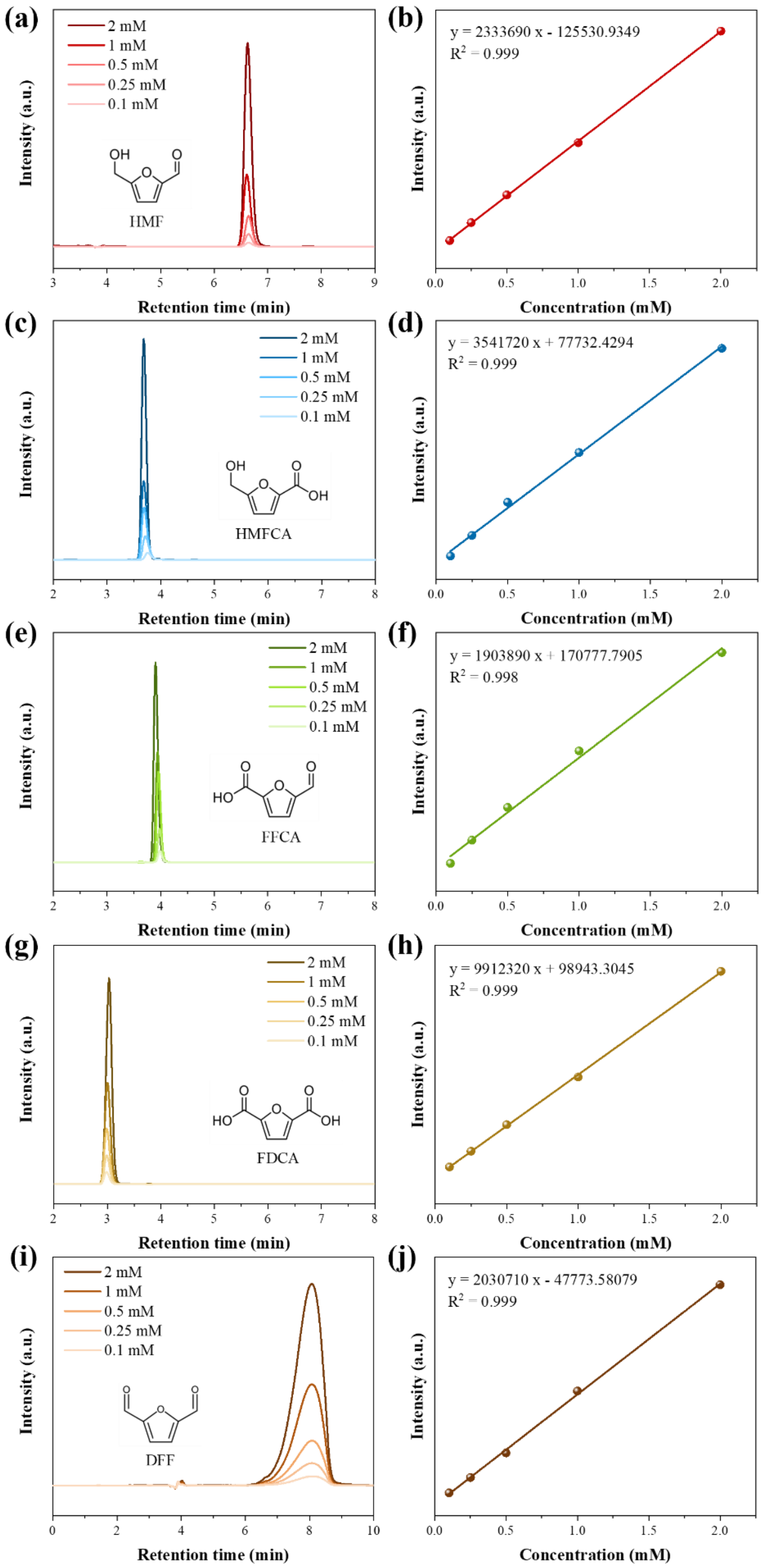

Figure S25. HPLC chromatograms and the corresponding standard curves of (a, b) HMF; (c, d) HMFCA; (e, f) FFCA; (g, h) FDCA; and (i, j) DFF.

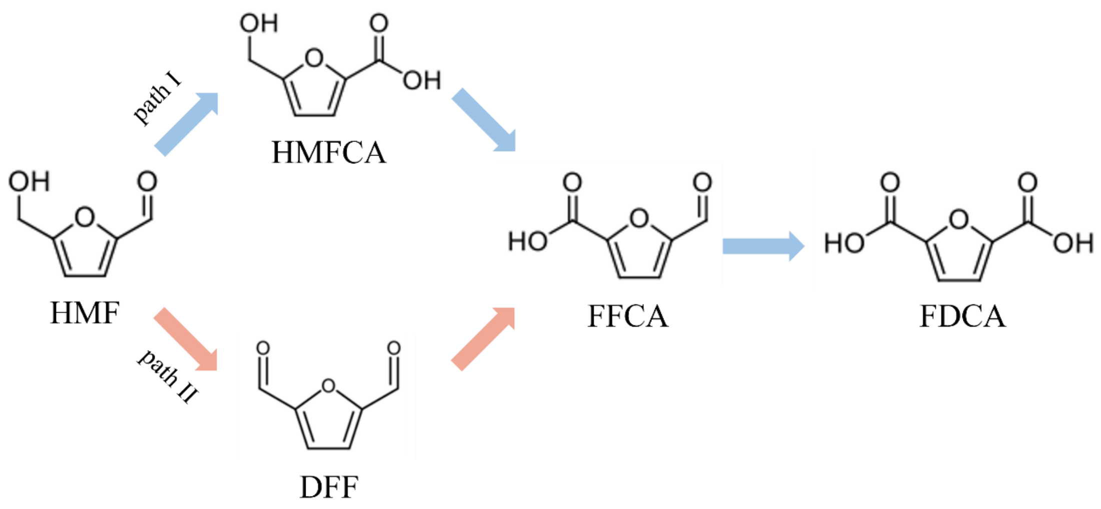

Figure S26. Two possible reaction paths for HMF electrooxidation to FDCA.

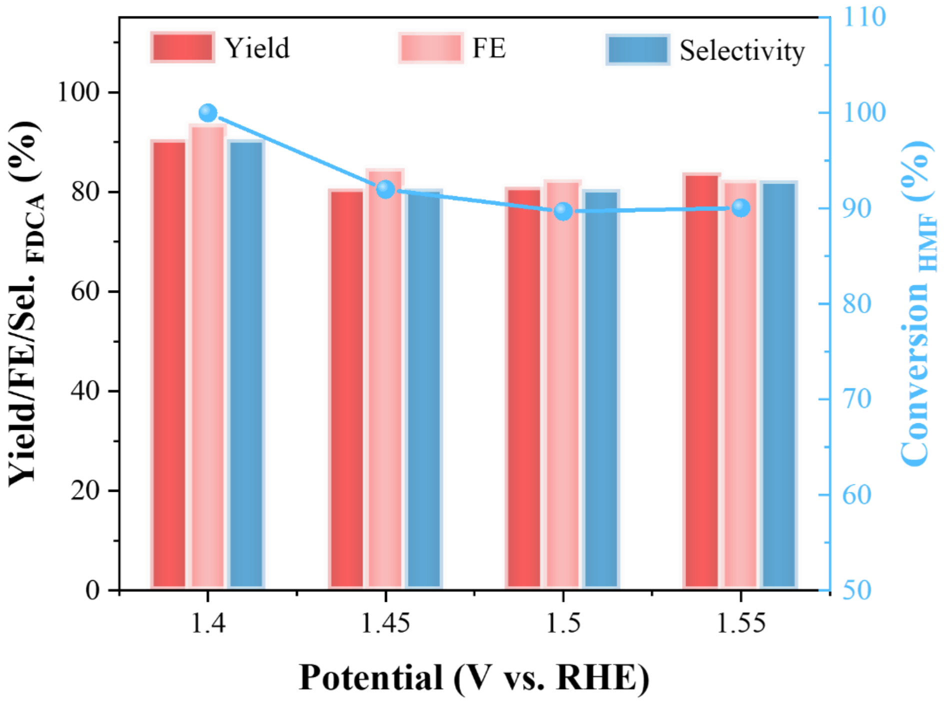

Figure S27. FE_{FDCA}, $yield_{FDCA}$, $selectivity_{FDCA}$ and HMF conversion of $Ni(OH)_2$@NF at different potentials in a 10 mM HMF solution.

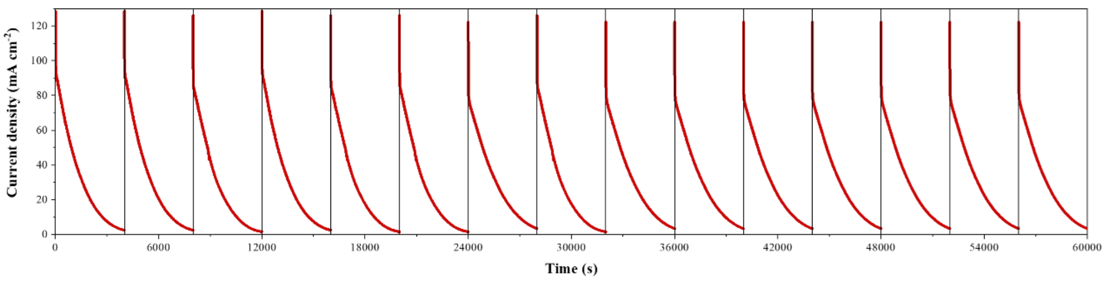

Figure S28. i-t curves of the Cu-Ni(OH)$_2$@NF sample at 1.45 V in consecutive cycles.

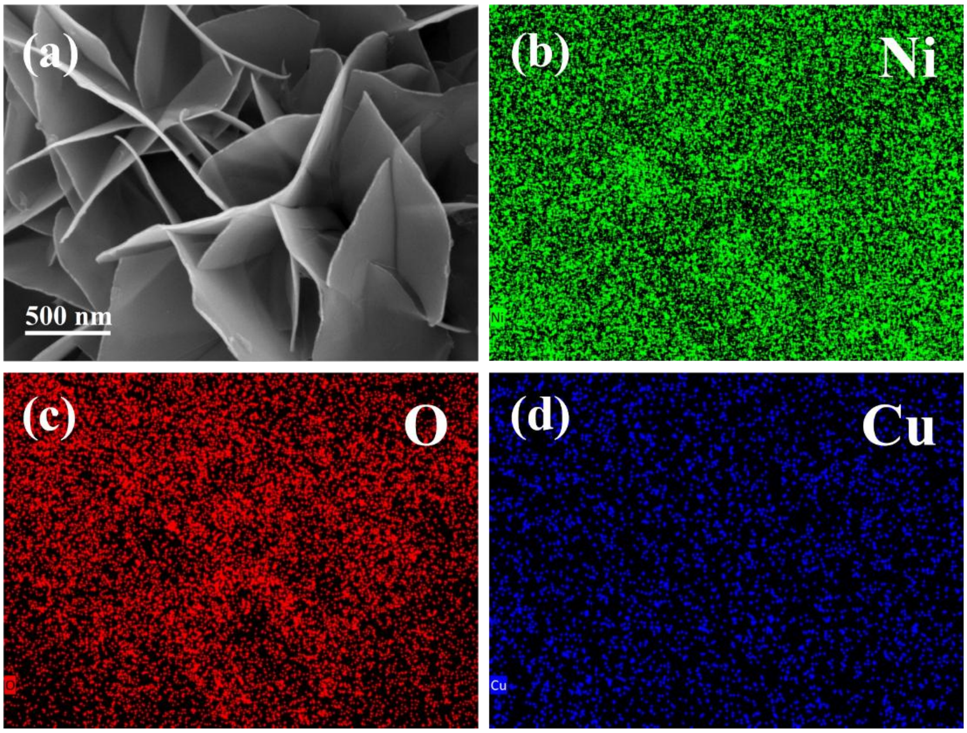

Figure S29. Morphology characterization of the recovered Cu-Ni(OH)$_2$@NF catalyst after long-term HMFOR tests. (a) SEM image; (b-d) EDS elemental mappings of (b) Ni; (c) O; and (d) Cu.

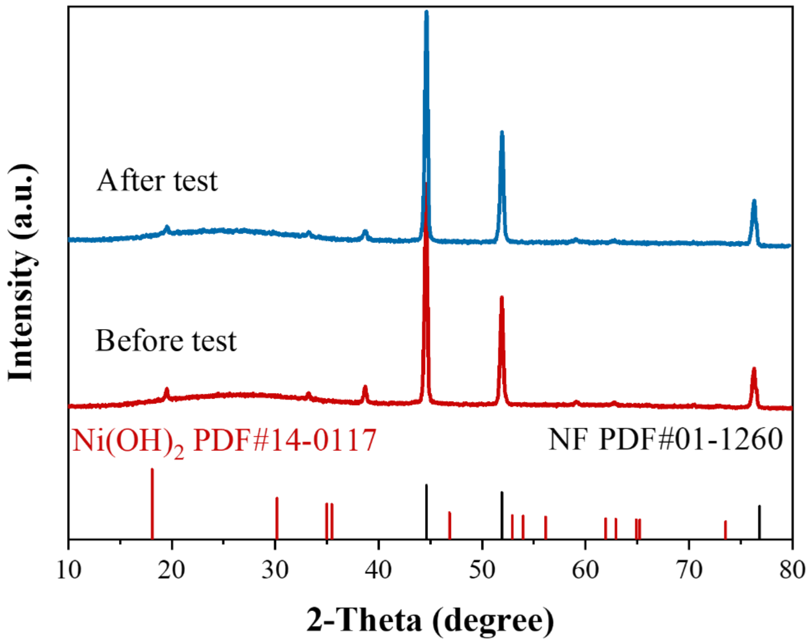

Figure S30. XRD spectra of Cu-$Ni(OH)_2$@NF before and after long-term HMFOR testing.

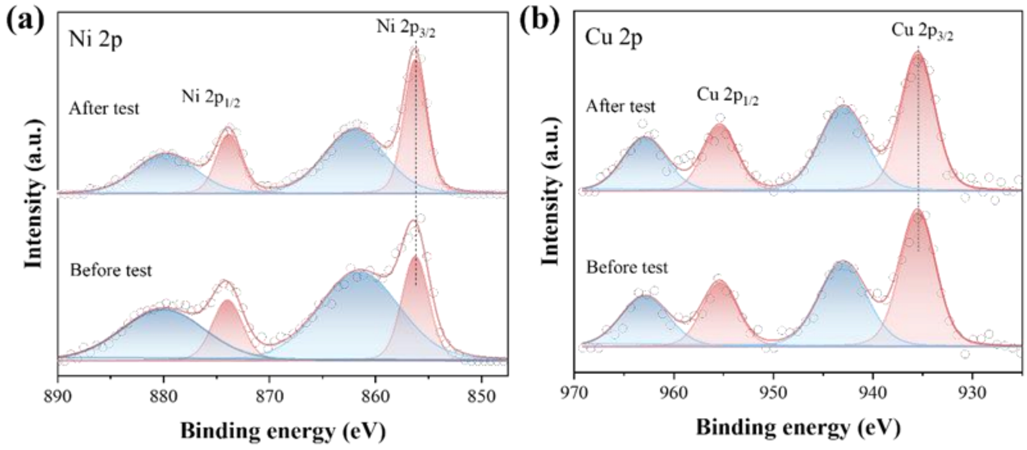

Figure S31. High-resolution XPS spectra of (a) Ni 2p and (b) Cu 2p in Cu-$Ni(OH)_2$@NF before and after long-term HMFOR testing.

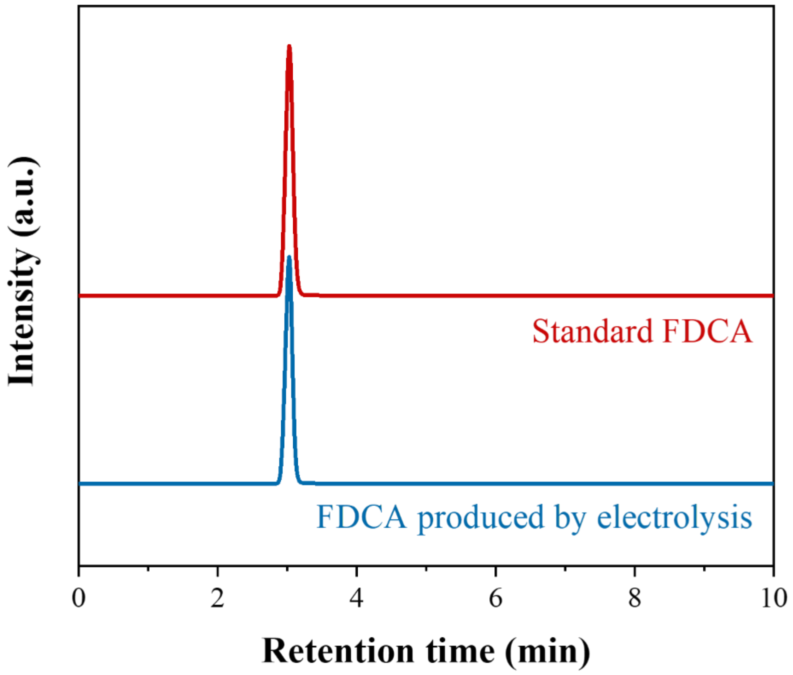

Figure S32. HPLC chromatograms of commercial FDCA and FDCA produced by electrolysis.

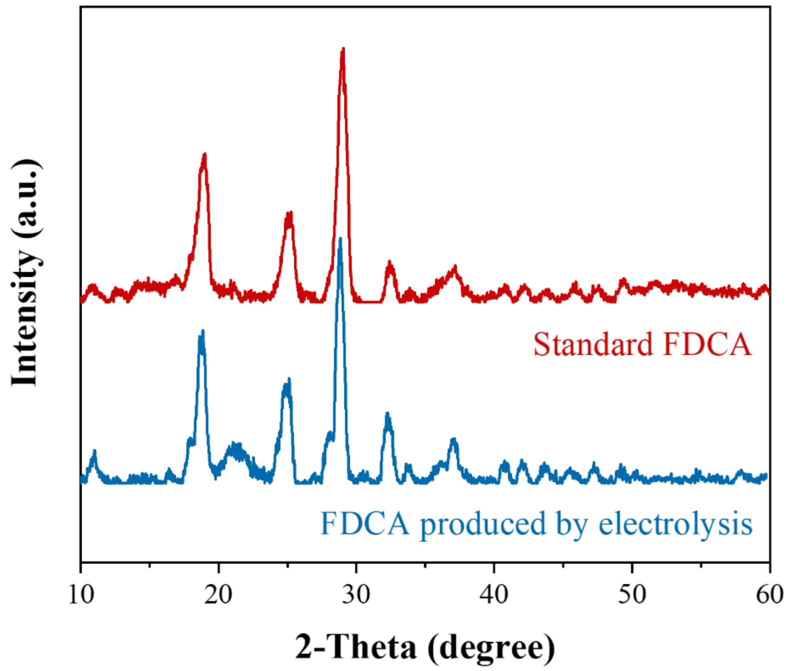

Figure S33. XRD spectra of commercial FDCA and FDCA produced by electrolysis.

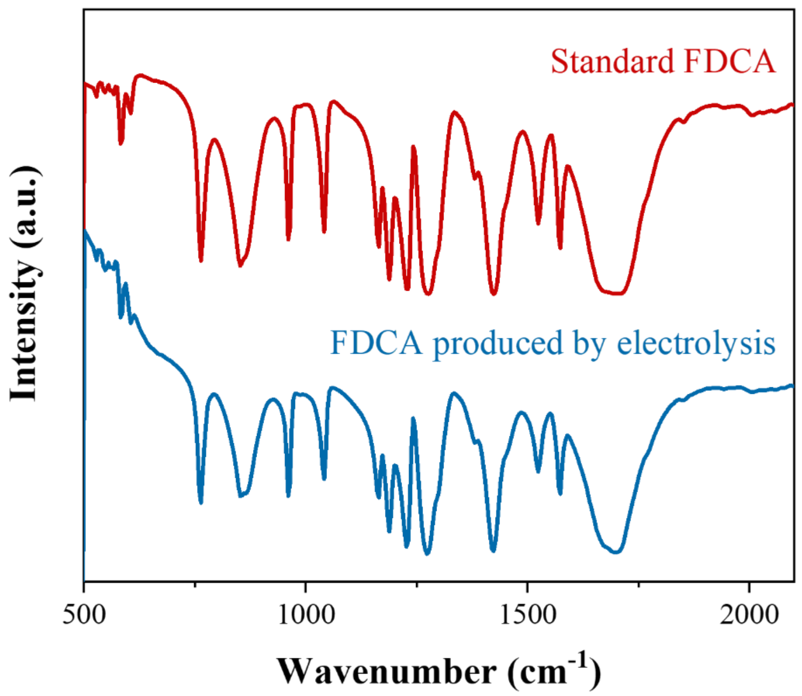

Figure S34. FT-IR spectra of commercial FDCA and FDCA produced by electrolysis.

Table S1. ICP-OES results of Cu-Ni$(OH)_2$@NF.

Catalyst	Ni wt%	O wt%	Cu wt%
Cu-Ni$(OH)_2$@NF	67.15	28.48	4.37

Table S2. Comparison of HMF electrooxidation performances ever reported.

Catalyst	C_{HMF} (mM)	Potential (V vs. RHE)	Current density (mA cm^{-2})	References
Cu-Ni(OH)$_2$@NF	20	1.50	372	**This work**
Cu-Ni(OH)$_2$@NF	50	1.60	1200	**This work**
(FeCrCoNiCu)$_3$O$_4$	50	1.65	30	*Angew. Chem. Int. Ed.* **2021**, 60, 20253.
Pt/CuO@CF	50	1.55	347	*Adv. Mater*. **2025**, 37, 202417684.
CuO-PdO	50	1.42	20	*Adv. Mater.* **2022**, 34, e2204089.
Rh-O$_5$/Ni(Fe)	50	1.40	100	*J. Am. Chem. Soc.* **2023**, 145, 17577.
Ni$_3$S$_2$/NiO$_x$	20	1.50	366	*Adv. Mater.* **2023**, n/a, 2304133.
Co$_{0.4}$NiS@NF	50	1.43	500	*Adv. Sci.* **2022**, 9, 2200957.
NiCu NTs	20	1.60	156	*J. Mater. Chem. A.* **2022**, 10, 10181.
Ni$_{0.9}$Cu$_{0.1}$(OH)$_2$	5	1.68	30	*J. Mater. Chem. A.* **2021**, 9, 9685.
Ni$_3$P-Cu$_3$P/CF	10	1.60	300	*Nano Energy.* **2024**, 127, 109727.
NiOOH/Ni(OH)$_2$/Ni	10	1.50	60	*Adv. Mater.* **2025**, 37, 202419050.

Table S3. The related EIS fitted parameters of the $Ni(OH)_2$@NF catalyst for OER.

Potential	R_s	CPE_1-T	CPE_1-P	R_{ct}	CPE_2-T	CPE_1-P	R_p
1.1 V	0.4166	0.02011	0.5863	3.869	0.03193	0.8814	3808
1.2 V	0.4251	0.03294	0.5682	3.14	0.064	0.8529	1859
1.3 V	0.5266	0.1745	0.4203	2.032	3.449	0.8923	11.35
1.35 V	0.5667	0.6741	0.421	0.7072	2.835	0.7022	13.81
1.40 V	0.4619	0.9149	0.429	0.6879	1.808	0.8174	30.64
1.45 V	0.4573	0.41	0.5115	0.2485	1.136	0.7016	42.99
1.50 V	0.4739	0.3683	0.5201	0.2383	0.889	0.7073	8.766
1.55 V	0.5193	0.3173	0.5015	0.2783	0.7041	0.7137	3.164
1.60 V	0.5749	0.3591	0.4775	0.3249	0.6318	0.7048	1.418

Table S4. The related EIS fitted parameters of the $Ni(OH)_2$@NF catalyst for HMFOR.

Potential	R_s	CPE_1-T	CPE_1-P	R_{ct}	CPE_2-T	CPE_1-P	R_p
1.1 V	0.4974	0.03119	0.5478	3.972	0.05037	0.9058	1085
1.2 V	0.547	0.02957	0.6078	2.899	0.08131	0.7789	427.5
1.3 V	0.5198	0.241	0.5084	1.961	0.312	0.4471	24.61
1.35 V	0.5374	0.4115	0.5276	0.9328	2.551	0.7243	3.561
1.40 V	0.51	0.9887	0.3836	1.609	2.226	0.6846	10.17
1.45 V	0.5144	0.7796	0.4316	1.23	4.733	1.042	5.918
1.50 V	0.511	0.6699	0.4243	0.8617	1.233	0.7469	12.34
1.55 V	0.5374	0.3715	0.4885	0.5543	0.9773	0.7984	3.883
1.60 V	0.5968	0.24	0.527	0.445	0.7653	0.7547	1.75

Table S5. The related EIS fitted parameters of the Cu-Ni$(OH)_2$@NF catalyst for OER.

Potential	R_s	CPE_1-T	CPE_1-P	R_{ct}	CPE_2-T	CPE_1-P	R_p
1.1 V	0.4862	0.07671	0.5182	7.192	0.03156	0.9688	1694
1.2 V	0.5104	0.06318	0.5671	4.316	0.04351	0.8863	1040
1.3 V	0.5076	0.1271	0.5191	3.469	0.1243	0.8308	323.4
1.35 V	0.5019	0.2211	0.4868	2.727	0.3059	0.8044	68.92
1.40 V	0.5014	0.3928	0.6127	0.9422	0.4204	0.5066	51
1.45 V	0.5004	0.8535	0.4588	0.4025	0.9375	0.6419	17.76
1.50 V	0.4885	1.362	0.4006	0.2575	0.9119	0.6961	6.559
1.55 V	0.5302	1.047	0.3985	0.3763	0.8014	0.7772	2.381
1.60 V	0.537	1.214	0.2827	0.3522	0.7441	0.7653	1.392

Table S6. The related EIS fitted parameters of the Cu-Ni$(OH)_2$@NF catalyst for HMFOR.

Potential	R_s	CPE_1-T	CPE_1-P	R_{ct}	CPE_2-T	CPE_1-P	R_p
1.1 V	0.4895	0.04958	0.5944	2.52	0.03348	0.8838	698.7
1.2 V	0.4651	0.1066	0.4961	2.087	0.1057	1.069	88.74
1.3 V	0.4945	0.1261	0.5601	1.137	0.196	0.7365	12.38
1.35 V	0.4817	0.009175	1.226	0.6224	0.3495	0.4735	5.76
1.40 V	0.4895	0.2009	0.6604	0.4752	1.062	0.4638	2.55
1.45 V	0.5258	0.27	0.6124	0.3942	2.045	0.8137	2.302
1.50 V	0.4666	0.1422	0.7086	0.6873	0.9901	0.5236	8.442
1.55 V	0.4908	0.7654	0.4706	0.4139	1.022	0.8316	2.627
1.60 V	0.5897	0.1767	0.6941	0.141	0.5739	0.7961	1.785

Table S7. ICP-OES results of $Cu\text{-}Ni(OH)_2$@NF after long-term HMFOR testing.

Catalyst	Ni wt%	O wt%	Cu wt%
$Cu\text{-}Ni(OH)_2$@NF	66.21	29.81	3.98
